# Sex-dependent locus coeruleus vulnerability in Alzheimer’s disease: gut dysbiosis as a driver and probiotic intervention as rescue

**DOI:** 10.1186/s13293-026-00834-8

**Published:** 2026-01-27

**Authors:** Hannah M. Stapleton, Dayanne S. Borges, Erasmo B. S. M. Trindade, Qi Yuan

**Affiliations:** 1 Biomedical Sciences, Faculty of Medicine, Memorial University of Newfoundland, St. John’s, NL A1B 3V6 Canada; 2 Postgraduate Program in Nutrition, Federal University of Santa Catarina, Florianopolis, 88040970 Santa Catarina Brazil

**Keywords:** Locus coeruleus, Norepinephrine, Gut microbiome, Dysbiosis, Sex differences, Alzheimer’s disease, Probiotics, Inflammation, BBB

## Abstract

Alzheimer’s disease (AD) displays striking sex differences in incidence, progression, and resilience, yet the mechanisms that drive female-biased vulnerability remain incompletely understood. Emerging evidence indicates that gut dysbiosis, increasingly prevalent with ageing, acts as a systemic amplifier of neuroinflammation, vascular instability, and metabolic dysfunction. Here, we synthesize converging findings linking gut microbial alterations to noradrenergic pathology in the locus coeruleus (LC), one of the earliest brain regions affected in AD. We outline how dysbiosis-associated inflammatory signaling, including endotoxin exposure and impaired vagal-neuroimmune regulation, targets LC circuits. In parallel, disruptions in microbial metabolite pathways involving short-chain fatty acids, bile acids, and tryptophan metabolism further promote oxidative stress, tau phosphorylation, and neurodegeneration. We further argue that sex-dependent differences in immune reactivity, autonomic regulation, and hormonal transitions, particularly peri- and post-menopausal estrogen decline, render female LC neurons uniquely vulnerable to microbial and inflammatory perturbation. We propose a mechanistic framework in which gut dysbiosis destabilizes LC integrity through parallel immune-vascular, metabolite, endocrine, and vagal neural pathways, thereby accelerating cognitive decline and AD progression. Understanding how microbial signaling intersects with sex biology and neuromodulatory circuitry may reveal therapeutic windows for early intervention, including microbiome restoration, neuromodulatory tuning, and sex-specific metabolic targeting.

## Introduction

Alzheimer’s disease (AD) pathogenesis unfolds along trajectories shaped by both intrinsic neural circuit vulnerability and systemic modifiers such as sex hormones and the gut microbiome. Among neuromodulatory hubs, the locus coeruleus (LC), the brain’s principal source of norepinephrine (NE), is uniquely positioned as a convergence site where metabolic, inflammatory, and hormonal stressors interact to influence cognitive resilience and degeneration [1]. Compelling evidence indicates that LC neurons accumulate pretangle tau decades before cortical pathology emerges, linking early noradrenergic dysfunction to prodromal cognitive decline [2–4]. Notably, LC architecture, stress responsivity, and immune coupling exhibit pronounced sex differences [5, 6], suggesting that biological sex and hormonal milieu critically shape LC susceptibility cross aging and disease.

Here, we propose a *Gut–LC–Sex Convergence Hypothesis*, in which sex-biased LC vulnerability in AD arises from the coordinated interaction of circulatory, immune, hormonal, vagal, and metabolic pathways, with gut dysbiosis acting as a systems-level amplifier. This integrative framework accounts for a female-biased breach of LC resilience, particularly during peri- and post-menopausal transitions, and distinguishes the present review from prior accounts that consider only isolated components of this network. The following sections synthesize preclinical and clinical evidence supporting this model and highlight emerging therapeutic opportunities targeting this nexus in AD.

## Sex-dependent LC vulnerability in AD: preclinical and clinical findings

Across species, the LC-NE system calibrates arousal, attention, synaptic plasticity, microglial tone, and vascular dynamics, precisely the domains that drift early in AD [7–11]. Neuropathology places pretangle tau in LC neurons decades before clinical symptoms [2, 12–14]. Converging human imaging [15–18] and postmortem work [19–21] links LC integrity to cognition and AD progression, positioning LC measures as candidate prodromal biomarkers. Preclinical models reinforce causality: LC insults worsen amyloid deposition, neuroinflammation, network instability, and behavioral decline [22–25]; conversely, preserving LC‑NE signaling can rescue cognition and synaptic plasticity [26, 27].

Rodent work consistently identifies sex differences in LC macro‑ and micro‑architecture and stress coupling. Females typically show larger LC volume, greater neuron number, and more elaborate dendrites [6, 28–31]. Estrogen can up‑regulate tyrosine hydroxylase and boost NE synthesis [32, 33], while corticotropin‑releasing factor (CRF) signaling is biased toward prolonged Gs‑coupling in females, reducing receptor internalization and heightening stress sensitivity [34–37]. These features may enhance performance in optimal contexts yet increase metabolic and oxidative load during chronic adversity, amplifying vulnerability with aging and disease. Transcriptomic [38] and proteomic data [39] point to female‑specific stress pathways in LC neurons.

In LC-focused tauopathy models, including fibril seeding in PS19 and P301 lines [40, 41], LC-targeted pseudophosphorylated tau (htauE14) [42], and TgF344-AD models [27], tau pathology arises early, producing axonal degeneration, circuit dysfunction, and learning impairments prior to widespread cortical tangle formation. While earlier studies often pooled sexes, recent LC-specific htauE14 work reveals marked sex-divergent molecular and inflammatory responses, aligning with a female-biased vulnerability to tau stress [43, 44]. In this pretangle model, males show robust upregulation of NE biosynthetic genes, indicative of a compensatory response aimed at maintaining noradrenergic signaling in the face of tau-induced axonal degeneration. In contrast, females exhibit broader shifts in metabolic and developmental programs that may reflect alternative adaptive strategies shaping differential resilience [44].

Neuromelanin‑sensitive MRI have transformed LC research. Across aging and AD, lower LC integrity relates to greater tau burden and worse cognition [16–18]. However, sex effects remain under‑sampled or inconsistently analyzed: several cohorts report no macrostructural sex differences [45, 46], whereas others show lower [15] or higher [47, 48] neuromelanin-sensitive LC signal intensity in females compared to males. Additionally, studies have reported elevated functional connectivity [49, 50] and increased metabolism [51] in older females during the preclinical stages of AD, suggesting compensatory mechanisms that may confer resilience and slow disease progression. Methodological variation, limited stratification by menopausal status or hormone therapy, and disease stage mixing (preclinical, MCI, AD stages) likely blur sex‑dependent signals. Taken together, these findings position the LC as an early, behaviorally relevant node in human AD, underscoring the need for sex-aware longitudinal imaging integrated with fluid and genetic risk markers.

### Gut dysbiosis in ageing and Alzheimer’s disease: sex-dependent patterns

Ageing and AD are consistently associated with a shift in gut microbial composition characterized by reduced diversity, loss of short-chain fatty acid (SCFA) producers (e.g., *Bifidobacterium*, *Faecalibacterium*), and expansion of pro-inflammatory taxa such as *Escherichia* and broader *Proteobacteria* [52–54]. These changes weaken intestinal barrier integrity, increasing translocation of lipopolysaccharides (LPS) and other microbial products into the circulation, where they amplify systemic cytokine load (IL-6, TNF-α, IL-1β) and activate downstream immune signaling [52, 53, 55]. In the brain, this inflammatory pressure promotes blood-brain barrier (BBB) leakage, microglial priming, astrocytic reactivity, β-amyloid aggregation, tau phosphorylation, and ultimately neurodegeneration [4, 53, 55].

In parallel with the humoral immune route, gut dysbiosis also influences the brain through neural, metabolic, and endocrine channels [56]. One major communication pathway is vagal afferent signaling, which rapidly conveys microbial and visceral information directly to brainstem nuclei. Dysbiosis additionally modifies the production of microbial metabolites, including SCFAs, tryptophan-kynurenine derivatives, and bile acids, all of which can regulate neuronal excitability, microglial activation, and metabolic homeostasis. A further layer of communication arises from enteroendocrine hormones such as glucagon-like peptide-1 (GLP-1), ghrelin, and cortisol, which act on hypothalamic and limbic targets or modulate synaptic plasticity and neuroimmune tone [53, 56, 57].

Preclinical and clinical studies converge on the same pattern: dysbiosis tracks with cognitive decline, neuroinflammation, and proteinopathy, while interventions that restore microbial balance such as probiotics, prebiotics, antibiotics, or fecal transfer, can improve cognition and reduce neuroinflammatory markers [53–55]. In animal models, disrupted microbial balance increases Aβ deposition, tau phosphorylation, neuroinflammation, and cognitive impairment [58, 59], potentially driven by cGAS-STING and NLRP3 inflammasome activation [59–63]. Fecal microbiota transplantation (FMT) from AD patients into mice inducing Aβ/tau aggregation and synaptic injury, accompanied by elevated IL-1β, IL-18, and TNF-α [63, 64]. Human evidence aligns with this pattern. Clinical studies report that individuals with dysbiosis show lower microbial diversity, metabolic imbalance, and cognitive deterioration, and that FMT from healthy donors improves cognitive scores and alters metabolic signatures in AD and mild cognitive impaired (MCI) patients [65–67].

Therapeutically, microbiome-directed interventions reliably reverse pathology: probiotics reduce Aβ production and microglial activation in amyloid precursor protein (APP) knock-in mice [68] and ameliorate cognitive impairment via AKT/GSK-3β signalling modulation in aged AD-prone mice [69], while clinical probiotic supplementation improves cognition, reduces inflammatory markers, and promotes oxidative balance in mild-to-moderate AD [70, 71], with no effects in advanced stages [72]. Additionally, preclinical antibiotic depletion of gut flora reduces amyloid burden and slows disease progression [73], further demonstrating that manipulating the microbiome can modify AD trajectory rather than simply correlate with it. Together, these studies reinforce the interpretation that dysbiosis is mechanistically linked to both cognitive and pathological burden in AD, and that restoring microbial ecology can rescue cognition, reduce neuroinflammatory tone, and attenuate proteinopathy, especially at early or preclinical stages.

Despite broad parallels in how dysbiosis interfaces with AD, biological sex introduces divergence at nearly every level of this axis. Male and female microbiota differ across life in taxonomic composition, metabolite profiles, bile-acid metabolism, vagal sensitivity, and inflammatory tone [74–79]. Sex hormones such as estrogen and progesterone influence immune function and microglial reactivity. These hormonal signals fluctuate across the reproductive cycle, altering immune tone and microbial balance. With peri- and post-menopausal estrogen decline, these shifts become more pronounced, reconfiguring gut microbial communities and increasing vulnerability to AD [80]. Menopause-associated estrogen loss, in particular, reduces SCFA-producing taxa, elevates inflammatory signaling, and disrupts vascular-metabolic balance [81, 82], collectively creating a more permissive landscape for neurodegeneration. Additional modifiers, including diet, aging, physical activity, sleep, and geography, interact with sex to reshape the microbiota and influence disease trajectory [80]. Consistent with this, clinical cohorts show female-biased microbiome signatures tied to immune and metabolic status that parallel AD risk [52, 80, 83], while experimental models reveal heightened inflammatory reactivity and increased susceptibility to neuromodulatory stress in females, especially under conditions of noradrenergic decline [38, 85–87].

**Fig. 1:**
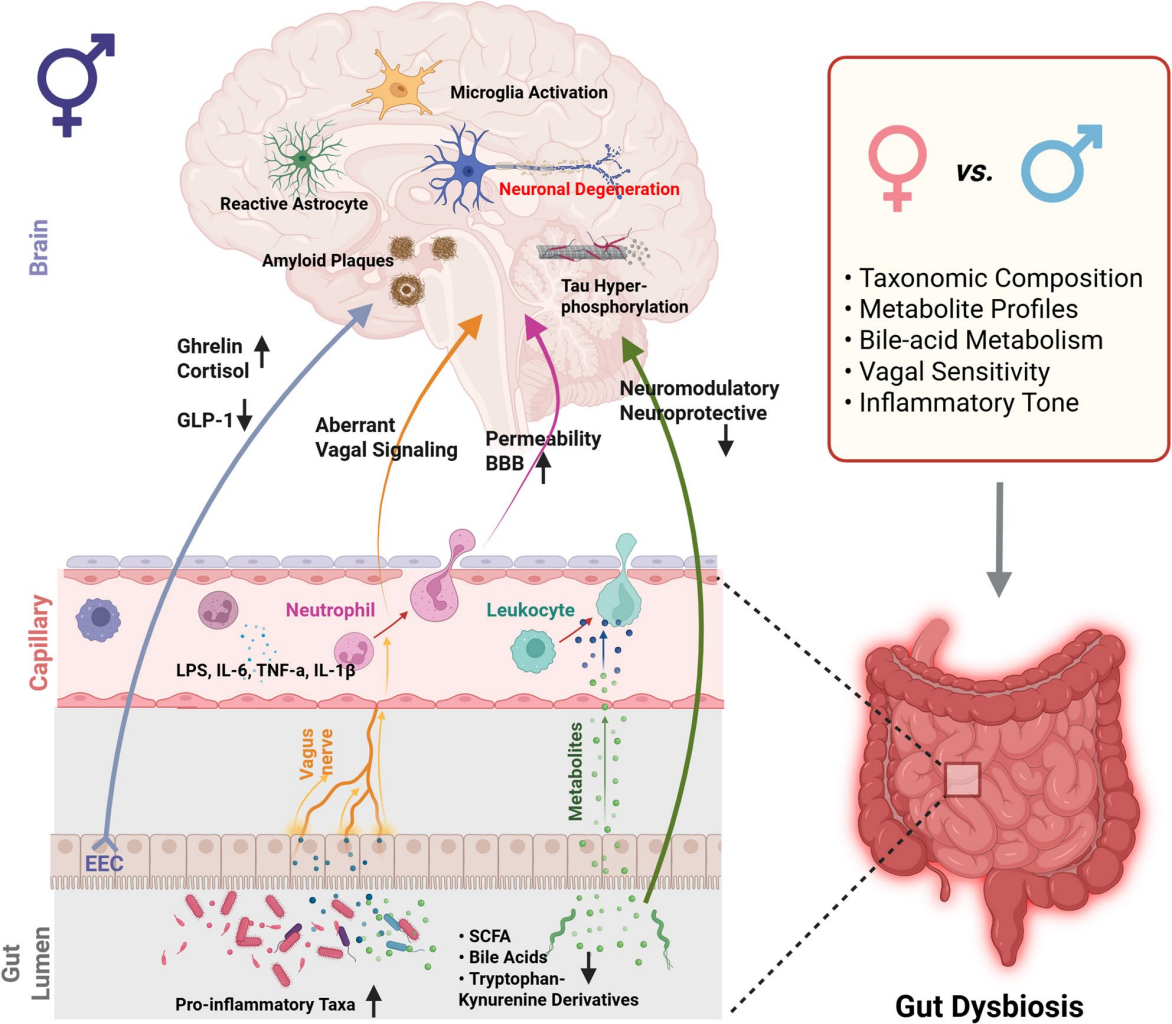
Sex-dependent pathways linking gut dysbiosis to neurodegenerative processes in the brain. ⚥ indicates gut-brain pathways shared by females and males (left), whereas ♀ and ♂ denote sex-specific aspects of gut microbiome dynamics Gut dysbiosis shifts microbial composition and disrupts normal metabolite production. Beneficial metabolites such as SCFAs, bile acids, and tryptophan-kynurenine derivatives decline, while pro-inflammatory taxa increase. Together, these alterations influence the brain through several interconnected pathways. Microbial products (e.g., LPS, IL-6, TNF-α, IL-1β) cross gut and vascular barriers, enter circulation, and promote leukocyte and neutrophil activation that contributes to blood-brain barrier permeability and inflammatory signaling. Dysbiosis also disrupts enteroendocrine communication (GLP-1, ghrelin, cortisol) and modulates vagal afferent pathways, leading to aberrant neural signaling toward brainstem and forebrain regions. These converging inputs promote microglial activation, reactive astrocytosis, tau hyperphosphorylation, amyloid accumulation, and neuronal degeneration. Sex differences influence every stage of this gut-brain axis, including microbial taxonomic profiles, metabolite balance, bile-acid dynamics, vagal sensitivity, and inflammatory tone, resulting in distinct trajectories of neuroimmune stress and neurodegenerative vulnerability in females and males. Generated with BioRender.com

Together, these findings position dysbiosis as a sex-modulated upstream driver of neurodegenerative vulnerability (Fig. 1). As Sect. “Why the locus coeruleus is particularly susceptible to dysbiosis: Sex-ependent mechanistic pathways and evidence for intervention” details, this imbalance does not act in isolation, it converges on the LC, a neuromodulatory hub exceptionally sensitive to inflammatory, metabolic, and hormonal perturbation. We propose that age- and menopause-linked microbial shifts form a systemic pressure point that disproportionately destabilizes noradrenergic homeostasis in females, accelerating LC degeneration and cognitive decline in AD.

### Why the locus coeruleus is particularly susceptible to dysbiosis: sex-dependent mechanistic pathways and evidence for intervention

The gut-brain axis forms a bidirectional communication network linking intestinal microbes with central neuromodulatory systems through neural, immune, endocrine, and metabolic routes [56, 57]. Within this framework, the LC stands out not simply as a relay, but as a high-gain integrator of visceral and inflammatory signals, owing to its dense capillary supply, proximity to permeable brainstem regions, and reciprocal connections with autonomic nuclei [1, 87]. High oxidative demand and an extensive projection architecture further heighten its sensitivity to peripheral perturbations, particularly those arising from microbial imbalance.

Emerging evidence suggests that dysbiosis may not only accompany aging and AD progression but operate upstream, priming LC physiology for degeneration. Microbiota shifts during dysbiosis reduce SCFA-mediated trophic support, elevate cytokine-driven inflammatory tone, and disrupt vagal and hypothalamic-pituitary axis (HPA) dynamics, creating multiple biological access points through which gut signals destabilize LC homeostasis. These pathways can activate microglia, intensify oxidative stress, and disturb noradrenergic output, effectively lowering the threshold for tau phosphorylation and synaptic loss.

**Fig. 2:**
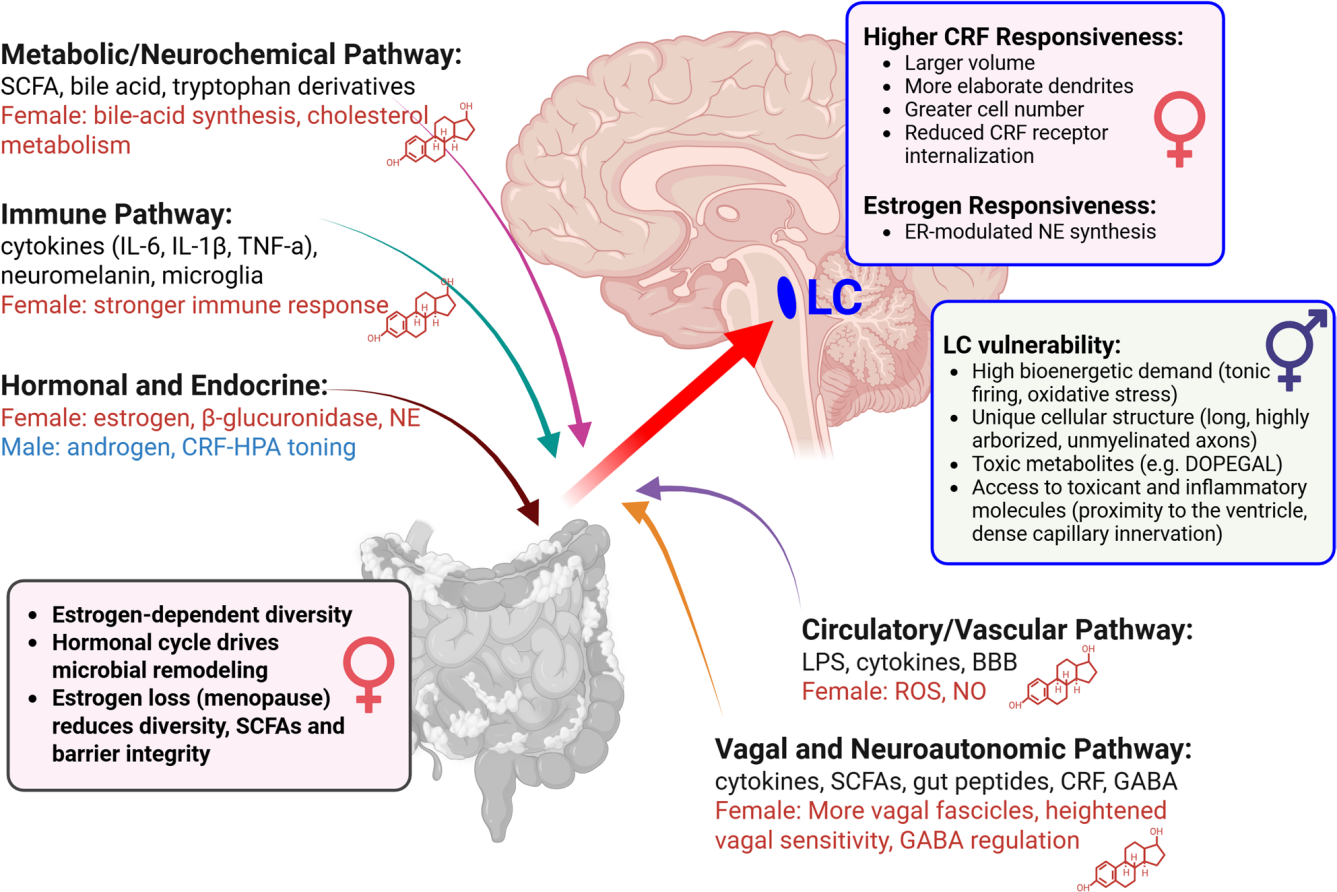
Sex-dependent gut-brain pathways converging on LC vulnerability in Alzheimer’s disease. ⚥ indicates features of LC vulnerability shared by females and males, whereas ♀ denotes female-specific gut microbiome features and heightened CRF responsiveness in the LC. Estrogen molecular structure denotes estrogen-modulated processes The gut microbiome communicates with the brain through circulatory, immune, hormonal, vagal, and metabolic pathways that collectively converge on the LC, each subject to sex-dependent modulation. Estrogen promotes microbial diversity, epithelial barrier integrity, and short-chain fatty acid (SCFA) production, whereas its decline with menopause reduces SCFAs, increases gut permeability, and heightens inflammatory tone. Females show stronger immune activation, greater ROS/NO vascular signaling, and higher vagal sensitivity with enhanced GABAergic modulation; estrogen also regulates β-glucuronidase activity, norepinephrine (NE) synthesis, and bile-acid metabolism. LC neurons exhibit female-biased CRF sensitivity and estrogen-dependent NE regulation, increasing vulnerability as hormonal support diminishes. Because LC neurons have high oxidative demand, long unmyelinated axons, and close vascular apposition, they readily encounter cytokines, LPS, and toxic catecholamine metabolites. Together, these sex- and hormone-dependent factors link gut dysbiosis and endocrine decline to LC oxidative stress and neurodegeneration, positioning the LC as a central convergence node and a promising target for microbiome-based interventions. Abbreviations: LC, locus coeruleus; NE, norepinephrine; SCFA, short-chain fatty acid; CRF, corticotropin-releasing factor; HPA, hypothalamic-pituitary-adrenal axis; ROS, reactive oxygen species; NO, nitric oxide; LPS, lipopolysaccharide; DOPEGAL, 3,4-dihydroxyphenylglycolaldehyde. Generated with BioRender.com

Taken together, gut dysbiosis emerges as a vulnerability amplifier for the LC, with susceptibility shaped by sex-dependent differences in immune responsivity, steroid hormone signaling, and neuromodulatory feedback. The following subsections delineate five mechanistic routes through which microbial imbalance can influence LC integrity including circulatory, immune, hormonal, vagal, and metabolic/neurochemical, highlighting points of sex-specific divergence (Fig. 2).

### Circulatory and vascular pathway

Situated adjacent to the fourth ventricle and endowed with an exceptionally dense capillary network, the LC exhibits circumventricular organ-like features that increase its exposure to circulating mediators [88]. Indeed, perivascular noradrenergic terminals originating from the LC closely abut intracortical blood vessels and astrocytic processes, underscoring the LC’s intimate vascular-glial interface and vulnerability to systemic influences [89]. Gut dysbiosis elevates circulating LPS and inflammatory cytokines, inducing endotoxemia and compromising the BBB [90, 91]. LC neurons, characterized by thin myelination and perivascular positioning [89, 92, 93], are among the earliest neuronal populations to encounter these infiltrating molecules. Under physiological conditions, NE released from LC terminals maintains vascular tone and supports BBB stability [89, 94, 95]. Thus, LC degeneration diminishes NE-dependent vascular regulation and exacerbates barrier leakage and oxidative injury [22], creating a self-reinforcing cycle of vulnerability.

Estrogen confers vascular protection by enhancing nitric-oxide (NO) signaling and antioxidant defense [96–98], mitigating these insults in premenopausal females. With menopause, estrogen decline removes this protective buffer, therefore rendering the female LC more susceptible to vascular and oxidative stress. Consistent with this, both clinical and experimental models of estrogen deficiency demonstrate endothelial dysfunction, increased reactive oxygen species (ROS), and diminished NO bioavailability [99–101]. These impairments are reversed by estrogen replacement [102], indicating that hormonal loss alone is sufficient to precipitate vascular and oxidative injury. Notably, women also show a disproportionately higher mortality risk from cardiovascular disease [103], reflecting a broader trend in which sex-dependent biological mechanisms influence vulnerability and clinical outcomes across systems.

### Immune and inflammatory pathway

Gut dysbiosis compromises intestinal barrier integrity, permitting microbial antigens and metabolites to enter circulation and activate systemic immune cascades [56, 57]. Elevated cytokines such as IL-6 and TNF-α can penetrate permeable brain regions, stimulating microglial and astrocytic activation within the LC and initiating neuroinflammatory responses [4, 43]. The LC’s intrinsically high oxidative metabolism, driven by pacemaker-like autonomic firing [104], together with its neuromelanin accumulation, intensifies vulnerability to such insults [105]. Neuromelanin supports cellular resilience in healthy LC neurons, yet when released from degenerating cells it becomes pro-inflammatory, driving mitochondrial injury and elevating reactive oxygen species [106, 107]. Females typically mount stronger innate and adaptive immune responses, a process tightly modulated by estrogen and progesterone [108, 109]. With hormonal decline of peri- and menopause, anti-inflammatory signaling weakens and glial cells become primed for exaggerated reactivity. This sex-biased immune amplification further renders the LC as a particularly vulnerable target for inflammatory damage during aging and neurodegenerative disease.

### Hormonal and endocrine pathway

The gut microbiota-estrogen axis provides a metabolic-endocrine bridge between intestinal function and LC neuromodulatory regulation. Circulating estrogen levels are strongly shaped by microbial metabolism [110]. Following hepatic conjugation, estrogens are secreted into bile as biologically inactive glucuronide and sulfate conjugates [111, 112], then reactivated in the intestine where bacterial β-glucuronidases and hydroxysteroid dehydrogenases convert them back into biologically active hormones [113–115]. These recycled estrogens re-enter circulation and reach the brain, where LC-expressed ERα and ERβ [116] regulate noradrenergic tone - baseline and dynamic range of NE synthesis and release, by modulating tyrosine hydroxylase [32, 117] and catechol-O-methyltransferase [118]. Through this route, microbial metabolism of estrogens shapes LC bioenergetics, enhancing NE turnover, mitochondrial efficiency, and antioxidant capacity to support metabolic stability.

Dysbiosis that reduces estrogen-reactivating bacteria lowers circulating estradiol [119], weakening these neuroprotective mechanisms and blunting LC NE output. During menopause, the dual decline in microbial β-glucuronidase activity [120] and ovarian estrogen production acts synergistically to depress systemic estrogen levels, thereby compromising LC energy metabolism and resilience to oxidative stress.

Sex strongly shapes this endocrine coupling. In females, estrogen-driven feedback between the gut microbiota, circulation, and LC receptor signaling serves as a primary regulatory axis, whereas in males, androgenic pathways more prominently govern stress reactivity and metabolic homeostasis [121, 122]. Androgens such as testosterone and dihydrotestosterone engage receptors in hypothalamic, amygdalar, and brainstem autonomic nuclei to restrain CRF-dependent HPA activation and tonic sympathetic output [121, 123, 124], enhancing metabolic efficiency and reducing oxidative burden in LC neurons.

Under normal conditions, estrogenic signaling and microbiome-mediated estrogen recycling provide metabolic buffering within the LC, sustaining NE synthesis, mitochondrial efficiency, and antioxidant defenses even during periods of heightened stress [5, 125]. When either of these supports diminishes during peri- and menopause, dysbiosis, inflammation, or impaired enterohepatic cycling, metabolic stability may erode, potentially reducing the precision of LC firing and increasing the energetic cost of noradrenergic output. Reduced mitochondrial resilience would be expected to elevate ROS generation and lower recovery capacity, predisposing LC neurons to excitotoxic strain and degeneration [126]. This model offers a mechanistic basis for heightened LC stress sensitivity in peri-menopausal females, and for why gut dysbiosis may exacerbate noradrenergic vulnerability in AD.

Notably, metabolic dysregulation increases during peri- and post-menopause [127], and women with metabolic disorders show a higher likelihood of early menopausal onset [128, 129], highlighting a bidirectional feedback loop in which metabolic strain accelerates estrogen decline and reduced estrogen further destabilizes LC energetics. In this framework, microbial imbalance, hormonal depletion, and metabolic vulnerability do not act independently, but converge to produce a female-biased breach of LC resilience.

### Vagal and neuroautonomic pathway

The vagus nerve serves as the primary conduit linking gut physiology to the LC. Vagal afferents project to the nucleus tractus solitarius (NTS), which provides excitatory input to the LC and allows visceral states to modulate noradrenergic output [126, 130–132]. Vagus nerve stimulation preferentially activates myelinated vagal fibers, generating temporally patterned NTS bursts that induce phasic LC firing, enhancing signal-to-noise processing and adaptive arousal [126, 131]. In contrast, inflammatory cytokines activate hypothalamic CRF circuits via vagal afferent signaling [133], increasing CRF release onto LC neurons, a mechanism well-known to shift LC activity toward sustained tonic firing, hyperarousal, and stress responsivity [5, 84, 134, 135].

**Fig. 3:**
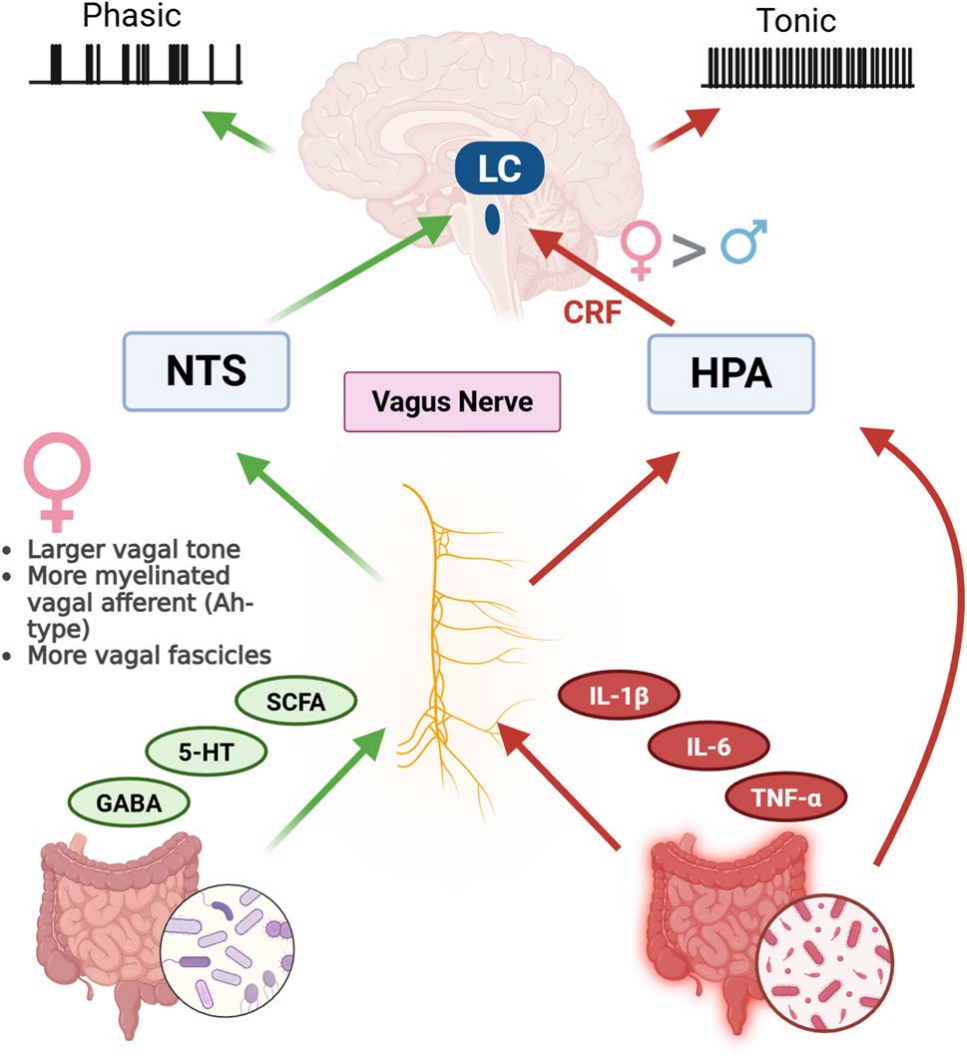
Sex-dependent vagal and HPA pathways linking gut dysbiosis to LC modulation. ♀ indicates female-specific vagal features (left) and female-biased LC responses to HPA-axis signaling (right) Gut-derived signals reach the LC through two major neural routes: the vagus nerve via the nucleus tractus solitarius (NTS; green arrows) and inflammatory-stress pathways via the hypothalamic-pituitary-adrenal (HPA) axis, which can also engage vagal pathways (red arrows). Under healthy conditions, microbial metabolites such as short-chain fatty acids (SCFAs), GABA, and serotonin (5-HT) enhance vagal afferent activity and support phasic LC firing. Dysbiosis reduces these metabolites while increasing pro-inflammatory cytokines (IL-1β, IL-6, TNF-α), which drive HPA activation and corticotrophin-releasing factor (CRF)-mediated tonic LC hyperactivity. Sex differences influence both pathways: females typically show higher vagal tone, more myelinated Ah-type vagal afferents, and greater vagal fascicle number, while LC neurons exhibit female-biased CRF sensitivity. Together, these factors create a sex-dependent bias in how gut-derived signals shape LC activity and stress responsivity. Generated with BioRender.com

Gut dysbiosis increases intestinal permeability and circulating IL-1β, TNF-α, and IL-6, thereby heightening vagal inflammatory signaling and HPA-CRF drive [133, 136, 137]. We propose that gut dysbiosis-associated inflammation preferentially engages HPA-CRF signaling, biasing LC neurons toward tonic, stress-like firing [138, 139]. This tonic mode competes with and can override the vagus nerve-NTS-LC pathway, which normally promotes phasic, stimulus-locked LC activity that supports attention, learning, and cognitive flexibility. Under conditions of sustained inflammatory or stress signaling, CRF-driven tonic firing dominates, reducing the influence of vagal phasic inputs and shifting LC output toward anxiety-related and maladaptive behavioral states (Fig. 3).

Dysbiosis also alters vagal tone through microbial metabolites and neurotransmitters, notably reducing SCFAs such as acetate, propionate, and butyrate [140]. SCFAs modulate vagal excitability both directly and indirectly: they act through GPR41/43 signaling on enteroendocrine and enteric neurons, stimulating the release of gut peptides such as GLP-1 and PYY that fine-tune vagal afferent firing and promote sustained suppression of peripheral inflammatory signaling [142–144]. In addition, SCFAs, particularly butyrate, enhance GABAergic signaling via histone deacetylase inhibition and increased GABA synthesis [144], effects that may contribute to the inhibitory tone regulating vagal output [141, 145]. When SCFA levels decline, this inhibitory control is weakened, producing erratic or tonic vagal firing which activates hypothalamus CRF neurons, consequently, sustained excitatory drive to the LC. Such dysregulation promotes LC overactivation, oxidative stress, and reduced cognitive function.

Sex potentially shapes vagal-LC coupling at both the afferent and central levels. In humans, premenopausal women exhibit higher cardiac vagal tone and more favorable autonomic balance than age-matched men, an effect that is closely linked to circulating estradiol and diminishes after menopause [146]. In rodents, vagal sensory neurons express estrogen receptors and show marked female-biased specializations: a myelinated Ah-type afferent population is more prevalent in females, its excitability is reduced by ovariectomy and restored by 17β-estradiol [147, 148]. Recent human anatomical work further suggests subtle sex dimorphism in the vagus nerve itself. Women display a greater number of fascicles, particularly in the right cervical vagus, potentially increasing channel capacity for visceral signaling [149]. On the efferent side, parasympathetic output to the gut arises from the dorsal motor nucleus of the vagus and nucleus ambiguus, where androgen receptors are abundant [150], indicating that androgens can modulate vagal motor neurons and metabolic/autonomic outflow.

In summary, these peripheral and brainstem differences interface with a sexually dimorphic LC (Fig. 3). LC-NE neurons in females differ in number, morphology, excitability, and stress responsivity from males [6]. Whole-brain rabies “projectome” mapping shows that female LC receives a relatively higher proportion of inputs from midbrain and hindbrain including autonomic regions, whereas male LC is more heavily innervated by cerebrum and interbrain structures [151]. In the context of gut dysbiosis, increased IL-1β, TNF-α, and IL-6 engage vagal afferents and hypothalamic CRF circuits, which in turn bias LC firing toward a tonic, stress-like mode [34]. When estrogen declines, female-biased vagal afferent sensitivity, reduced autonomic protection, and stronger hindbrain input to LC together provide a mechanistic framework for why dysbiosis and inflammatory load may more readily translate into LC hyperarousal, anxiety, and cognitive impairment in females than in males.

### Metabolic and neurochemical pathway

Microbial metabolites including SCFAs, bile acids, and tryptophan derivatives, act as neuromodulators influencing LC function. Under healthy conditions, SCFAs such as butyrate normally strengthen gut barrier integrity, suppress inflammation, and inhibit the expansion of harmful bacteria [152, 153]. When SCFAs are reduced, barrier function weakens and oxidative and immune stress increase [145], a pattern consistently observed in inflammatory bowel diseases [154].

The gut microbiome strongly shapes both tryptophan metabolism [155] and bile-acid metabolism [156], each of which contributes to neuromodulatory signaling in distinct ways [157, 158]. Disruptions in these metabolic pathways can alter LC activity through downstream mechanisms, including farnesoid X receptor (FXR)-mediated bile-acid signaling and serotonin-dependent pathways, that directly modulate autonomic tone [159, 160].

Under physiological conditions, bile acids act as signaling molecules that activate FXR and TGR5 receptors in peripheral and central autonomic circuits, helping maintain metabolic homeostasis and balanced autonomic output [161, 162]. Gut dysbiosis disrupts bile-acid metabolism, lowering secondary bile-acid production [163] and weakening TGR5 signaling [164]. Because TGR5 is selective for secondary bile acids [165], this impairment heightens inflammation [166] and contributes to broader metabolic dysfunction [167].

Similarly, gut microbial imbalances shift tryptophan metabolism away from the serotonin pathway toward the kynurenine pathway, reducing central serotonin availability [168] and generating toxic kynurenine metabolites that promote neuroinflammation [169]. Because serotonin normally provides inhibitory modulation of LC neurons through 5-HT₂A receptors [170], this shift disinhibits LC firing and promotes a tonic, stress-like state. Together, bile-acid and tryptophan dysregulation reinforce autonomic imbalance and noradrenergic overactivation, exacerbating LC vulnerability to metabolic and inflammatory stress.

Sex differences further shape these metabolic pathways. In humans, males generally maintain larger bile-acid pools [171], whereas in mice young females show the larger pools [172, 173]. Estrogen regulates bile-acid synthesis and FXR-dependent feedback control of cholesterol and lipid metabolism [174, 175], in part by suppressing CYP7A1 and thereby maintaining a smaller, tightly regulated bile-acid pool in females [176]. After menopause, declines in both estrogen and microbial SCFA production weaken this metabolic buffering, reducing antioxidant capacity and mitochondrial efficiency [177, 178]. The combined loss of hormonal and microbial regulation heightens redox imbalance and catecholaminergic stress, could contribute to the increased LC vulnerability observed in females.

### The locus coeruleus: convergence node and probiotic rescue target

Together, these five pathways define the LC as a hub of convergence where circulatory, immune, hormonal, autonomic, and metabolic signals intersect (Fig. 2; Table 1). Under healthy conditions, NE output, estrogen signaling, and microbial metabolites maintain vascular, immune, and stress balance. Dysbiosis dismantles this homeostasis, provoking systemic inflammation, hormonal imbalance, and altered vagal signaling, that collectively overburden LC neurons. Female-specific traits including stronger immune reactivity, estrogen-linked vascular control, and higher vagal tone, magnify this coupling, producing a sex-biased vulnerability axis that aligns with women’s higher AD risk and earlier LC decline [3].

**Table 1:** Gut-Brain communication pathways influencing LC vulnerability and sex modulation

Pathway	Gut-Brain Communication during Dysbiosis	Impact on Locus Coeruleus (LC)	Sex Differences/Modulation	Key Mediators
1. Circulatory/Vascular	Gut dysbiosis elevates circulating LPS and cytokines (IL-6, TNF-α, IL-1β), leading to systemic inflammation and BBB disruption.	LC, near the fourth ventricle with dense capillaries, is directly exposed to circulating toxins; NE loss further weakens vascular tone and BBB integrity.	Estrogen promotes NO signaling and antioxidant defense; its loss post-menopause increases vascular fragility and female LC vulnerability.	LPS, IL-6, TNF-α, ROS, NO, NE, estrogen
2. Immune/Inflammatory	Microbial antigens cross a leaky gut barrier, triggering systemic immune activation. Cytokines activate LC microglia and astrocytes.	Chronic inflammation and oxidative load overwhelm LC’s metabolic demand; neuromelanin shifts from antioxidant to pro-oxidant, causing mitochondrial injury.	Females exhibit stronger immune reactivity; estrogen decline augments glial activation and cytokine release.	IL-6, IL-1β, TNF-α, neuromelanin, estrogen, microglia
3. Hormonal/Endocrine	Gut microbiota regulates estrogen metabolism through β-glucuronidase and hydroxysteroid dehydrogenase activity; dysbiosis lowers estradiol.	Reduced estrogen decreases NE synthesis (via tyrosine hydroxylase) and antioxidant protection, lowering LC resilience.	In females, microbial and ovarian estrogen decline converge after menopause; males rely more on androgen pathways.	β-glucuronidase, estradiol, ERα/ERβ, TH, COMT
4. Vagal/Neuroautonomic	Vagal afferents from gut → NTS → LC; dysbiosis alters gut peptides and cytokines, aberrantly activating vagal input to the HPA axis.	Shifts LC firing from adaptive phasic to stress-like tonic mode, impairing attention and stress regulation.	Females have more vagal fascicles and estrogen receptors in vagal nuclei; fluctuating GABA tone during cycle increases visceral sensitivity.	IL-1β, TNF-α, CCK, GLP-1, ghrelin, PYY, CRF, estrogen
5. Metabolic/Neurochemical	Microbial metabolites (SCFAs, bile acids, tryptophan derivatives) influence energy metabolism and neurotransmitter synthesis.	SCFA loss increases oxidative stress; altered bile-acid and tryptophan pathways disturb catecholaminergic stability in LC.	Males have larger bile-acid pools; estrogen modulates FXR/TGR5 signaling and its decline removes metabolic buffering in females.	Butyrate, bile acids, FXR, TGR5, serotonin, kynurenine, estrogen

BBB: blood–brain barrier; CCK: cholecystokinin; COMT: catechol-O-methyltransferase; CRF: corticotropin-releasing factor; E2: estradiol (estrogen); ERα/ERβ: estrogen receptor alpha/beta; FXR: farnesoid X receptor; GLP-1: glucagon-like peptide-1; IL-1β, IL-6: interleukin-1 beta, interleukin-6; LC: locus coeruleus; LPS: lipopolysaccharide; NE: norepinephrine; NO: nitric oxide; NTS: nucleus tractus solitarius; PYY: peptide YY; ROS: reactive oxygen species; SCFAs: short-chain fatty acids; TH: tyrosine hydroxylase; TNF-α: tumor necrosis factor alpha; TGR5: Takeda G protein-coupled receptor 5

Empirical data support the reversibility of this vulnerability. In the LC-targeted pretangle-tau rat model by Flynn et al. (2025), chronic synbiotic (probiotic + prebiotic) treatment enhanced microbial diversity, enriched *Bifidobacterium Lactis*, and boosted SCFA-producing microbiome. These changes lowered systemic cytokine levels, and reversed LC microgliosis and BBB leakage. Behaviorally, treated animals displayed improved spatial learning and reduced anxiety, reflecting normalized LC-cortical NE transmission. At the molecular level, notably, females exhibited activation of resilience pathways, such as GSK-3β inhibition in the hippocampus, a major LC target [43].

These findings demonstrate that LC sensitivity to dysbiosis is dynamic and therapeutically modifiable. Restoring gut-brain homeostasis stabilizes LC signaling and protects noradrenergic integrity. Translationally, this supports a precision-medicine strategy that integrates microbiome modulation, vagus-based neuromodulation, and hormonal support to delay LC-linked cognitive decline, particularly in women who are predisposed to AD [179].

## Translational implications and future directions

Emerging technologies now allow the integration of LC-specific imaging, systemic biomarkers, and microbiome profiling to define prodromal windows of noradrenergic vulnerability. Longitudinal studies combining neuromelanin-sensitive LC-MRI with plasma/CSF inflammatory signatures and microbial diversity metrics should incorporate sex-based stratification across menopausal state, hormone therapy, and contraceptive exposure. Such multidimensional modeling will help resolve how hormonal transitions interact with microbial composition to shape LC decline and may reveal precision windows for targeted intervention.

In preclinical systems, mechanistic studies should pair LC-centric physiological and molecular readouts including firing dynamics, NE release, BBB integrity, microglial activation, and transcriptomic remodeling, with gut-brain interventions such as probiotics, prebiotics, dietary modulation, and vagus nerve stimulation. Explicit evaluation of sex x treatment interactions will be critical for uncovering differential resilience mechanisms. The LC-targeted pretangle-tau model is especially well-suited for pre-clinical therapeutic testing, enabling multimodal outcome mapping from ultrastructure and immunohistochemistry to kinase signaling pathways linking microbial ecology to LC cellular stress.

Looking forward, personalized intervention strategies should anticipate sex-specific therapeutic responses across both pharmacological and microbial domains. Precision probiotic strain selection or dosing, sex-differentiated neuromodulatory targets (e.g., anti-CRF approaches or adrenergic receptor tuning), and combinatorial regimens integrating microbiome modulation, lifestyle factors, and neuromodulatory therapies may together provide a framework to buffer LC vulnerability across aging trajectories.

### Outstanding questions

- Which microbial metabolites (SCFAs, bile acids, tryptophan derivatives) most strongly couple to LC stress biology, and do their brain entry routes differ by sex and age?
- Could integrating neuromelanin-sensitive LC-MRI with plasma metabolomics yield a practical, minimally invasive biomarker panel for early detection of LC decline and for stratifying women by menopausal status and metabolic risk?
- In probiotic/prebiotic responders, are benefits mediated primarily by vascular/immune normalization, vagal recalibration, or intracellular kinase shifts, and do these pathways differ by sex?

## Conclusions

Together, the evidence reviewed here suggests that gut dysbiosis is not a passive correlate of AD but an upstream biological driver capable of shaping neural vulnerability. Microbial imbalance, reduced SCFA production, altered metabolite profiles, and heightened inflammatory tone converge with vagal, endocrine, and metabolic signaling to destabilize LC homeostasis, a critical early site of AD pathology. We highlight that these pathways interact strongly with biological sex: females exhibit greater inflammatory reactivity, distinct microbial trajectories across ageing, and experience abrupt estrogen loss, all of which amplify the impact of dysbiosis on LC integrity and cognitive decline.

Importantly, the relationship appears reversible. Microbiome restoration through diet, probiotics, prebiotics, FMT, or metabolic modulation improves cognition and reduces neuroinflammation in animal models and emerging clinical trials, underscoring the gut as a modifiable therapeutic target. We propose that future work must test whether preserving microbial diversity can stabilize LC function, slow tau propagation, and delay disease onset, particularly in women, who bear disproportionate AD risk. A mechanistic focus on the gut-LC-sex axis may open new intervention windows long before traditional clinical decline emerges.

## Data Availability

Data sharing is not applicable to this article as no datasets were generated or analyzed during the current study.

## Abbreviations

AD: Alzheimer’s Disease
APP: Amyloid precursor protein
BBB: Blood-brain barrier
CRF: Corticotropin-releasing factor
FMT: Fecal microbiota transplantation
FXR: Farnesoid X receptor
GLP-1: Glucagon-like peptide-1
HPA: Hypothalamic-pituitary axis
LC: Locus coeruleus
LPS: Lipopolysaccharides
MCI: Mild cognitive impaired
NE: Norepinephrine
NO: Nitric-oxide
NTS: Nucleus tractus solitarius
ROS: Reactive oxygen species
SCFA: Short-chain fatty acid

## References

[1] Matchett BJ, Grinberg LT, Theofilas P, Murray ME. The mechanistic link between selective vulnerability of the locus coeruleus and neurodegeneration in Alzheimer’s disease. Acta Neuropathol. 2021;141(5):631–50. 10.1007/s00401-020-02248-1. 33427939 10.1007/s00401-020-02248-1 PMC8043919

[2] Braak H, Thal DR, Ghebremedhin E, Del Tredici K. Stages of the pathologic process in Alzheimer disease: age categories from 1 to 100 years. J Neuropathol Exp Neurol. 2011;70(11):960–9. 10.1097/NEN.0b013e318232a379. 22002422 10.1097/NEN.0b013e318232a379

[3] Luckey AM, Robertson IH, Lawlor B, Mohan A, Vanneste S. Sex differences in locus coeruleus: a heuristic approach that may explain the increased risk of Alzheimer’s disease in females. J Alzheimer’s Disease: JAD. 2021;83:2505–22. 10.3233/JAD-210404. 10.3233/JAD-210404 34334399

[4] Yuan Q, Omoluabi T, Hannam BF. Shifting focus to preclinical stages: locus coeruleus Tau pathology as a driver and therapeutic target in Alzheimer’s disease. Neural Regen Res. 2025. 10.4103/NRR.NRR-D-25-00140. 40637566 10.4103/NRR.NRR-D-25-00140 PMC13211810

[5] Bangasser DA, Wiersielis KR, Khantsis S. Sex differences in the locus coeruleus-norepinephrine system and its regulation by stress. Brain Res. 2016;1641:177–88. 10.1016/j.brainres.2015.11.021. 26607253 10.1016/j.brainres.2015.11.021 PMC4875880

[6] Bangasser DA, Zhang X, Garachh V, Hanhauser E, Valentino RJ. Sexual dimorphism in locus coeruleus dendritic morphology: a structural basis for sex differences in emotional arousal. Physiol Behav. 2011;103:3–4. 10.1016/j.physbeh.2011.02.037. 10.1016/j.physbeh.2011.02.037 PMC3081983 21362438

[7] Poe GR, Foote S, Eschenko O, Johansen JP, Bouret S, Aston-Jones G, et al. Locus coeruleus: a new look at the blue spot. Nat Rev Neurosci. 2020;21(11):644–59. 10.1038/s41583-020-0360-9. 32943779 10.1038/s41583-020-0360-9 PMC8991985

[8] Sara SJ. The locus coeruleus and noradrenergic modulation of cognition. Nat Rev Neurosci. 2009;10(3):211–23. 10.1038/nrn2573. 19190638 10.1038/nrn2573

[9] Aston-Jones G, Cohen JD. Adaptive gain and the role of the locus coeruleus-norepinephrine system in optimal performance. J Comp Neurol. 2005;493:1:99–110. 10.1002/cne.20723. 16254995 10.1002/cne.20723

[10] Weinshenker D. Long road to ruin: noradrenergic dysfunction in neurodegenerative disease. Trends Neurosci. 2018;41:4. 10.1016/j.tins.2018.01.010. 29475564 10.1016/j.tins.2018.01.010 PMC5878728

[11] Mather M, Harley CW. The locus coeruleus: essential for maintaining cognitive function and the aging brain. Trends Cogn Sci. 2016;20:3214–26. 10.1016/j.tics.2016.01.001. 10.1016/j.tics.2016.01.001 PMC4761411 26895736

[12] Braak H, Del Tredici K. The pathological process underlying alzheimer’s disease in individuals under Thirty. Acta Neuropathol. 2011;121:2171–81. 10.1007/s00401-010-0789-4. 10.1007/s00401-010-0789-4 21170538

[13] Braak H, Del Tredici K. Where, when, and in what form does sporadic Alzheimerʼs disease begin?: Curr Opin Neurol. 2012;25(6):708–14. 10.1097/WCO.0b013e32835a3432. 23160422 10.1097/WCO.0b013e32835a3432

[14] Braak H, Del Tredici K. Neuroanatomy and pathology of sporadic alzheimer’s disease. Adv Anat Embryol Cell Biol. 2015;215:1–162. http://www.ncbi.nlm.nih.gov/pubmed/25920101. 25920101

[15] Clewett DV, Lee TH, Greening S, Ponzio A, Margalit E, Mather M. Neuromelanin marks the spot: identifying a locus coeruleus biomarker of cognitive reserve in healthy aging. Neurobiol Aging. 2016;37:117–26. 10.1016/j.neurobiolaging.2015.09.019. 26521135 10.1016/j.neurobiolaging.2015.09.019 PMC5134892

[16] Dahl MJ, Mather M, Duzel S, Bodammer NC, Lindenberger U, Kuhn S, et al. Rostral locus coeruleus integrity is associated with better memory performance in older adults. Nat Hum Behav. 2019;3:11:1203–14. 10.1038/s41562-019-0715-2. 31501542 10.1038/s41562-019-0715-2 PMC7203800

[17] Bueicheku E, Diez I, Kim CM, Becker JA, Koops EA, Kwong K, et al. Spatiotemporal patterns of locus coeruleus integrity predict cortical Tau and cognition. Nat Aging. 2024. 10.1038/s43587-024-00626-y. 38664576 10.1038/s43587-024-00626-y PMC11108787

[18] Prokopiou PC, Engels-Dominguez N, Papp KV, Scott MR, Schultz AP, Schneider C, et al. Lower novelty-related locus coeruleus function is associated with Abeta-related cognitive decline in clinically healthy individuals. Nat Commun. 2022;13:11571. 10.1038/s41467-022-28986-2. 10.1038/s41467-022-28986-2 PMC8943159 35322012

[19] Kelly SC, He B, Perez SE, Ginsberg SD, Mufson EJ, Counts SE. Locus coeruleus cellular and molecular pathology during the progression of Alzheimer’s disease. Acta Neuropathol Commun. 2017;5(1):8. 10.1186/s40478-017-0411-2. 28109312 10.1186/s40478-017-0411-2 PMC5251221

[20] Wilson RS, Nag S, Boyle PA, Hizel LP, Yu L, Buchman AS, et al. Neural reserve, neuronal density in the locus ceruleus, and cognitive decline. Neurology. 2013;80(13):1202–8. 10.1212/WNL.0b013e3182897103. 23486878 10.1212/WNL.0b013e3182897103 PMC3691778

[21] Theofilas P, Ehrenberg AJ, Dunlop S, Di Lorenzo Alho AT, Nguy A, Leite REP, et al. Locus coeruleus volume and cell population changes during Alzheimer’s disease progression: a stereological study in human postmortem brains with potential implication for early-stage biomarker discovery. Alzheimers Dement. 2017;13(3):236–46. 10.1016/j.jalz.2016.06.2362. 27513978 10.1016/j.jalz.2016.06.2362 PMC5298942

[22] Kelly SC, McKay EC, Beck JS, Collier TJ, Dorrance AM, Counts SE. Locus coeruleus degeneration induces forebrain vascular pathology in a transgenic rat model of Alzheimer’s disease. J Alzheimer’s Disease: JAD. 2019;70:2371–88. 10.3233/JAD-190090. 10.3233/JAD-190090 PMC6929678 31177220

[23] Heneka MT, Ramanathan M, Jacobs AH, Dumitrescu-Ozimek L, Bilkei-Gorzo A, Debeir T, et al. Locus ceruleus degeneration promotes Alzheimer pathogenesis in amyloid precursor protein 23 transgenic mice. J Neurosci. 2006;26(5):1343–54. 10.1523/JNEUROSCI.4236-05.2006. 16452658 10.1523/JNEUROSCI.4236-05.2006 PMC6675491

[24] Hammerschmidt T, Kummer MP, Terwel D, Martinez A, Gorji A, Pape HC, et al. Selective loss of noradrenaline exacerbates early cognitive dysfunction and synaptic deficits in APP/PS1 mice. Biol Psychiatry. 2013;73(5):454–63. 10.1016/j.biopsych.2012.06.013. 22883210 10.1016/j.biopsych.2012.06.013 PMC4712953

[25] Kummer MP, Hammerschmidt T, Martinez A, Terwel D, Eichele G, Witten A, et al. Ear2 deletion causes early memory and learning deficits in APP/PS1 mice. J Neurosci. 2014;34(26):8845–54. 10.1523/JNEUROSCI.4027-13.2014. 24966384 10.1523/JNEUROSCI.4027-13.2014 PMC4147626

[26] Omoluabi T, Torraville SE, Maziar A, Ghosh A, Power KD, Reinhardt C, et al. Novelty-like activation of locus coeruleus protects against deleterious human pretangle tau effects while stress-inducing activation worsens its effects. Alzheimer’s & Dementia: Translational Research & Clinical Interventions. 2021;7(1):e12231. 10.1002/trc2.12231. 10.1002/trc2.12231 PMC8719346 35005208

[27] Rorabaugh JM, Chalermpalanupap T, Botz-Zapp CA, Fu VM, Lembeck NA, Cohen RM, et al. Chemogenetic locus coeruleus activation restores reversal learning in a rat model of Alzheimer’s disease. Brain. 2017;140(11):3023–38. 10.1093/brain/awx232. 29053824 10.1093/brain/awx232 PMC5841201

[28] Curtis AL, Bethea T, Valentino RJ. Sexually dimorphic responses of the brain norepinephrine system to stress and corticotropin-releasing factor. Neuropsychopharmacology. 2006;31(3):544–54. 10.1038/sj.npp.1300875. 16123744 10.1038/sj.npp.1300875

[29] Luque JM, de Blas MR, Segovia S, Guillamon A. Sexual dimorphism of the dopamine-β-hydroxylase-immunoreactive neurons in the rat locus ceruleus. Brain Res Dev Brain Res. 1992;67(2):211–5. 10.1016/0165-3806(92)90221-h. 1511516 10.1016/0165-3806(92)90221-h

[30] Pinos H, Collado P, Rodriguez-Zafra M, Rodriguez C, Segovia S, Guillamon A. The development of sex differences in the locus coeruleus of the rat. Brain Res Bull. 2001;56(1):73–8. 10.1016/s0361-9230(01)00540-8. 11604252 10.1016/s0361-9230(01)00540-8

[31] Babstock D, Malsbury CW, Harley CW. The dorsal locus coeruleus is larger in male than in female Sprague-Dawley rats. Neurosci Lett. 1997;224:3157–60. 10.1016/S0304-3940(97)13462-0. 10.1016/S0304-3940(97)13462-0 9131660

[32] Serova L, Rivkin M, Nakashima A, Sabban EL. Estradiol stimulates gene expression of norepinephrine biosynthetic enzymes in rat locus coeruleus. Neuroendocrinology. 2002;75(3):193–200. 10.1159/000048237. 11914591 10.1159/000048237

[33] Thanky NR, Son JH, Herbison AE. Sex differences in the regulation of tyrosine hydroxylase gene transcription by estrogen in the locus coeruleus of TH9-LacZ transgenic mice. Brain Res Mol Brain Res. 2002;104(2):220–6. 10.1016/s0169-328x(02)00383-2. 12225877 10.1016/s0169-328x(02)00383-2

[34] Valentino RJ, Bangasser DA. Sex-biased cellular signaling: molecular basis for sex differences in neuropsychiatric diseases. Dialogues Clin Neurosci. 2016;18(4):385–93. 10.31887/DCNS.2016.18.4/rvalentino. 28179810 10.31887/DCNS.2016.18.4/rvalentino PMC5286724

[35] Bangasser DA. Sex differences in stress-related receptors: “‘micro’” differences with “ ‘macro’” implications for mood and anxiety disorders. Biol Sex Differ. 2013;4(1):2. 10.1186/2042-6410-4-2. 23336736 10.1186/2042-6410-4-2 PMC3556142

[36] Bangasser DA, Curtis A, Reyes BA, Bethea TT, Parastatidis I, Ischiropoulos H, et al. Sex differences in corticotropin-releasing factor receptor signaling and trafficking: potential role in female vulnerability to stress-related psychopathology. Mol Psychiatry. 2010;15(9:877):96–904. 10.1038/mp.2010.66. 10.1038/mp.2010.66 PMC2935505 20548297

[37] Enman NM, Reyes BAS, Shi Y, Valentino RJ, Van Bockstaele EJ. Sex differences in morphine-induced trafficking of mu-opioid and corticotropin-releasing factor receptors in locus coeruleus neurons. Brain Res. 2019;1706:75–85. 10.1016/j.brainres.2018.11.001. 30391476 10.1016/j.brainres.2018.11.001

[38] Mulvey B, Bhatti DL, Gyawali S, Lake AM, Kriaucionis S, Ford CP, et al. Molecular and functional sex differences of noradrenergic neurons in the mouse locus coeruleus. Cell Rep. 2018;23(8):2225–35. 10.1016/j.celrep.2018.04.054. 29791834 10.1016/j.celrep.2018.04.054 PMC6070358

[39] Lee J, Wang ZM, Messi ML, Milligan C, Furdui CM, Delbono O. Sex differences in single neuron function and proteomics profiles examined by patch-clamp and mass spectrometry in the locus coeruleus of the adult mouse. Acta Physiol (Oxf). 2024;240(4):e14123. 10.1111/apha.14123. 38459766 10.1111/apha.14123 PMC11021178

[40] Ahnaou A, Walsh C, Manyakov NV, Youssef SA, Drinkenburg WH. Early electrophysiological disintegration of hippocampal neural networks in a novel locus coeruleus tau-seeding mouse model of Alzheimer’s disease. Neural Plast. 2019;2019:6981268. 10.1155/2019/6981268. 31285742 10.1155/2019/6981268 PMC6594257

[41] Iba M, McBride JD, Guo JL, Zhang B, Trojanowski JQ, Lee VM. Tau pathology spread in PS19 Tau transgenic mice following locus coeruleus (LC) injections of synthetic Tau fibrils is determined by the lc’s afferent and efferent connections. Acta Neuropathol. 2015;130:3349–62. 10.1007/s00401-015-1458-4. 10.1007/s00401-015-1458-4 PMC4545685 26150341

[42] Ghosh A, Torraville SE, Mukherjee B, Walling SG, Martin GM, Harley CW, et al. An experimental model of Braak’s pretangle proposal for the origin of Alzheimer’s disease: the role of locus coeruleus in early symptom development. Alzheimers Res Ther. 2019;11(1):59. 10.1186/s13195-019-0511-2. 31266535 10.1186/s13195-019-0511-2 PMC6607586

[43] Flynn CM, Omoluabi T, Janes AM, Rodgers EJ, Torraville SE, Negandhi BL, et al. Targeting early tau pathology: probiotic diet enhances cognitive function and reduces inflammation in a preclinical Alzheimer’s model. Alzheimers Res Ther. 2025;17(1):24. 10.1186/s13195-025-01674-1. 39827356 10.1186/s13195-025-01674-1 PMC11742226

[44] Omoluabi T, Hasan Z, Piche JE, Flynn ARS, Dore JJE, Walling SG, et al. Locus coeruleus vulnerability to tau hyperphosphorylation in a rat model. Aging Cell. 2025;24(3):e14405. 10.1111/acel.14405. 39520141 10.1111/acel.14405 PMC11896524

[45] Takahashi J, Shibata T, Sasaki M, Kudo M, Yanezawa H, Obara S, et al. Detection of changes in the locus coeruleus in patients with mild cognitive impairment and Alzheimer’s disease: high-resolution fast spin-echo T1-weighted imaging. Geriatr Gerontol Int. 2015;15(3):334–40. 10.1111/ggi.12280. 24661561 10.1111/ggi.12280 PMC4405055

[46] Calarco N, Cassidy CM, Selby B, Hawco C, Voineskos AN, Diniz BS et al. Associations between locus coeruleus integrity and diagnosis, age, and cognitive performance in older adults with and without late-life depression: an exploratory study. Neuroimage-Clin. 2022;36; doi:Artn 10318210.1016/J.Nicl.2022.103182. 10.1016/j.nicl.2022.103182 PMC9474922 36088841

[47] Riley E, Cicero N, Mabry SA, Swallow KM, Anderson AK, De Rosa E. Age-related differences in locus coeruleus intensity across a demographically diverse sample. Neurobiol Aging. 2025;150:122–31. 10.1016/j.neurobiolaging.2025.03.005. 40101307 10.1016/j.neurobiolaging.2025.03.005 PMC11981832

[48] Galgani A, Lombardo F, Frijia F, Scotto M, Tognoni G, Pavese N, et al. Locus coeruleus sexual dimorphism and its impact on cognitive impairment and cortical atrophy in Alzheimer’s disease. Neurodegener Dis. 2025;25(2):53–66. 10.1159/000544882. 40010329 10.1159/000544882

[49] Kilpatrick LA, Church A, Meriwether D, Mahurkar-Joshi S, Li VW, Sohn J et al. Differential brainstem connectivity according to sex and menopausal status in healthy male and female individuals. Biology of sex differences. 2025;16:1; doi:Artn 2510.1186/S13293-025-00709-4. 10.1186/s13293-025-00709-4 PMC12007138 40251694

[50] Um YH, Wang SM, Kang DW, Kim S, Lee CU, Kim D, et al. Sex-related disparities in the resting state functional connectivity of the locus coeruelus and salience network in preclinical Alzheimer’s disease. Int J Mol Sci. 2023. 10.3390/ijms242015092. 37894772 10.3390/ijms242015092 PMC10606651

[51] Koops EA, Dutta J, Hanseeuw BJ, Becker JA, Van Egroo M, Prokopiou PC, et al. Elevated locus coeruleus metabolism provides resilience against cognitive decline in preclinical Alzheimer’s disease. Alzheimers Dement. 2025;21(1):e14385. 10.1002/alz.14385. 39588792 10.1002/alz.14385 PMC11772725

[52] Hong SH, Roh HW, Nam YJ, Kim TW, Cho YH, Son SJ, et al. Age- and sex-specific gut microbiota signatures associated with dementia-related brain pathologies: an LEfSe-based metagenomic study. Brain Sci. 2025. 10.3390/brainsci15060611. 40563782 10.3390/brainsci15060611 PMC12191394

[53] Pavithra R, Kanimozhi NV, Sonali L, Suneetha C, Sukumar M. Unveiling role of gut microbiota in Alzheimer’s disease: mechanisms, challenges and future perspectives. Curr Alzheimer Res. 2025. 10.2174/0115672050403066250904112611. 40965029 10.2174/0115672050403066250904112611

[54] Zhou XP, Sun LB, Liu WH, Zhu WM, Li LC, Song XY et al. The complex relationship between gut microbiota and Alzheimer’s disease: A systematic review. Ageing Res Rev. 2025;104: 102637. 10.1016/j.arr.2024.102637 10.1016/j.arr.2024.102637 39662839

[55] Cryan JF, Dinan TG. Mind-altering microorganisms: the impact of the gut microbiota on brain and behaviour. Nat Rev Neurosci. 2012;13(10):701–12. 10.1038/nrn3346. 22968153 10.1038/nrn3346

[56] Cryan JF, O’Riordan KJ, Cowan CSM, Sandhu KV, Bastiaanssen TFS, Boehme M, et al. The microbiota-gut-brain axis. Physiol Rev. 2019;99(4):1877–2013. 10.1152/physrev.00018.2018. 31460832 10.1152/physrev.00018.2018

[57] Ohara TE, Hsiao EY. Microbiota-neuroepithelial signalling across the gut-brain axis. Nat Rev Microbiol. 2025;23(6):371–84. 10.1038/s41579-024-01136-9. 39743581 10.1038/s41579-024-01136-9

[58] Chen C, Ahn EH, Kang SS, Liu X, Alam A, Ye KQ. Gut dysbiosis contributes to amyloid pathology, associated with C/EBPβ/AEP signaling activation in Alzheimer’s disease mouse model. Science Advances. 2020;6:31; doi:ARTN eaba046610.1126/sciadv.aba0466. 10.1126/sciadv.aba0466 PMC7439296 32832679

[59] Zhao N, Chen X, Chen QG, Liu XT, Geng F, Zhu MM, et al. NLRP3-mediated autophagy dysfunction links gut microbiota dysbiosis to tau pathology in chronic sleep deprivation. Zool Res. 2024;45(4):857–74. 10.24272/j.issn.2095-8137.2024.085. 39004863 10.24272/j.issn.2095-8137.2024.085 PMC11298670

[60] Lei W, Cheng Y, Liu X, Gao J, Zhu Z, Ding W, et al. Gut microbiota-driven neuroinflammation in Alzheimer’s disease: from mechanisms to therapeutic opportunities. Front Immunol. 2025;16:1582119. 10.3389/fimmu.2025.1582119. 40642089 10.3389/fimmu.2025.1582119 PMC12241022

[61] Shmuel-Galia L, Humphries F, Lei X, Ceglia S, Wilson R, Jiang Z, et al. Dysbiosis exacerbates colitis by promoting ubiquitination and accumulation of the innate immune adaptor STING in myeloid cells. Immunity. 2021;54(6):1137–e538. 10.1016/j.immuni.2021.05.008. 34051146 10.1016/j.immuni.2021.05.008 PMC8237382

[62] Ising C, Venegas C, Zhang S, Scheiblich H, Schmidt SV, Vieira-Saecker A, et al. NLRP3 inflammasome activation drives tau pathology. Nature. 2019;575(7784):669–73. 10.1038/s41586-019-1769-z. 31748742 10.1038/s41586-019-1769-z PMC7324015

[63] Shen H, Guan Q, Zhang X, Yuan C, Tan Z, Zhai L, et al. New mechanism of neuroinflammation in Alzheimer’s disease: the activation of NLRP3 inflammasome mediated by gut microbiota. Prog Neuropsychopharmacol Biol Psychiatry. 2020;100:109884. 10.1016/j.pnpbp.2020.109884. 32032696 10.1016/j.pnpbp.2020.109884

[64] Xia Y, Xiao Y, Wang ZH, Liu X, Alam AM, Haran JP, et al. *Bacteroides fragilis* in the gut microbiomes of Alzheimer’s disease activates microglia and triggers pathogenesis in neuronal C/EBPβ transgenic mice. Nat Commun. 2023;14(1):5471. 10.1038/s41467-023-41283-w. 37673907 10.1038/s41467-023-41283-w PMC10482867

[65] Park SH, Lee JH, Shin J, Kim JS, Cha B, Lee S, et al. Cognitive function improvement after fecal microbiota transplantation in Alzheimer’s dementia patient: a case report. Curr Med Res Opin. 2021;37(10):1739–44. 10.1080/03007995.2021.1957807. 34289768 10.1080/03007995.2021.1957807

[66] Kim JS, Park H, Lee JH, Shin J, Cha B, Kwon KS, et al. Effect of altered gene expression in lipid metabolism on cognitive improvement in patients with Alzheimer’s dementia following fecal microbiota transplantation: a preliminary study. Ther Adv Neurol Disord. 2024;17:17562864231218181. 10.1177/17562864231218181. 38250318 10.1177/17562864231218181 PMC10799597

[67] Chen X, Zhang W, Lin Z, Zheng C, Chen S, Zhou H, et al. Preliminary evidence for developing safe and efficient fecal microbiota transplantation as potential treatment for aged related cognitive impairments. Front Cell Infect Microbiol. 2023;13:1103189. 10.3389/fcimb.2023.1103189. 37113132 10.3389/fcimb.2023.1103189 PMC10127103

[68] Abdelhamid M, Zhou C, Ohno K, Kuhara T, Taslima F, Abdullah M, et al. Probiotic bifidobacterium Breve prevents memory impairment through the reduction of both Amyloid-β production and microglia activation in APP Knock-In mouse. J Alzheimers Disease. 2022;85:4:1555–71. 10.3233/Jad-229022. 34958017 10.3233/JAD-215025 PMC8925106

[69] Qian XH, Chen SY, Tang HD. Multi-strain probiotics ameliorate Alzheimer ’ s-like cognitive impairment and pathological changes through the AKT/GSK-3 β pathway in senescence-accelerated mouse prone 8 mice. Brain Behav Immun. 2024;119:14–27. 10.1016/j.bbi.2024.03.031. 38548184 10.1016/j.bbi.2024.03.031

[70] Akhgarjand C, Vahabi Z, Shab-Bidar S, Etesam F, Djafarian K. Effects of probiotic supplements on cognition, anxiety, and physical activity in subjects with mild and moderate Alzheimer’s disease: A randomized, double-blind, and placebo-controlled study. Front Aging Neurosci. 2022;14. Artn 103249410.3389/Fnagi.2022.1032494. 10.3389/fnagi.2022.1032494 PMC9647197 36389063

[71] Akhgarjand C, Vahabi Z, Shab-Bidar S, Anoushirvani A, Djafarian K. The effects of probiotic supplements on oxidative stress and inflammation in subjects with mild and moderate Alzheimer’s disease: a randomized, double-blind, placebo-controlled study. Inflammopharmacology. 2024;32(2):1413–20. 10.1007/s10787-023-01427-2. 38319476 10.1007/s10787-023-01427-2

[72] Agahi A, Hamidi GA, Daneshvar R, Hamdieh M, Soheili M, Alinaghipour A et al. Does Severity of Alzheimer’s Disease Contribute to Its Responsiveness to Modifying Gut Microbiota? A Double Blind Clinical Trial. Front Neurol. 2018;9:662. 10.3389/Fneur.2018.00662. 10.3389/fneur.2018.00662 PMC6104449 30158897

[73] Minter MR, Zhang C, Leone V, Ringus DL, Zhang XQ, Oyler-Castrillo P, et al. Antibiotic-induced perturbations in gut microbial diversity influences neuro-inflammation and amyloidosis in a murine model of Alzheimer’s disease. Sci Rep. 2016. 10.1038/srep30028. 27443609 10.1038/srep30028 PMC4956742

[74] Sheng LL, Jena PK, Hu Y, Wan YJY. Age-specific microbiota in altering host inflammatory and metabolic signaling as well as metabolome based on the sex. Hepatobiliary Surg Nutr. 2021;10(1):31. 10.21037/hbsn-20-671. 33575288 10.21037/hbsn-20-671 PMC7867716

[75] Jasarevic E, Morrison KE, Bale TL. Sex differences in the gut microbiome-brain axis across the lifespan. Philos Trans R Soc Lond B Biol Sci. 2016;371(1688):20150122. 10.1098/rstb.2015.0122. 26833840 10.1098/rstb.2015.0122 PMC4785905

[76] Reichardt F, Lucas LN, Okyere L, Choi J, Amador-Noguez D, Gaulke CA, et al. Portal bile acid composition and microbiota along the murine intestinal tract exhibit sex differences in physiology. Gut Microbes. 2025;17(1):2540483. 10.1080/19490976.2025.2540483. 40760771 10.1080/19490976.2025.2540483 PMC12326574

[77] Santos-Marcos JA, Mora-Ortiz M, Tena-Sempere M, Lopez-Miranda J, Camargo A. Interaction between gut microbiota and sex hormones and their relation to sexual dimorphism in metabolic diseases. Biology of sex differences. 2023;14:1; doi:ARTN 410.1186/s13293-023-00490-2. 10.1186/s13293-023-00490-2 PMC9903633 36750874

[78] Shobeiri P, Kalantari A, Teixeira AL, Rezaei N. Shedding light on biological sex differences and microbiota-gut-brain axis: a comprehensive review of its roles in neuropsychiatric disorders. Biology of sex differences. 2022;13:1; doi:Artn 1210.1186/S13293-022-00422-6. 10.1186/s13293-022-00422-6 PMC8949832 35337376

[79] Kim YS, Unno T, Kim BY, Park MS. Sex differences in gut microbiota. World J Mens Health. 2020;38(1):48–60. 10.5534/wjmh.190009. 30929328 10.5534/wjmh.190009 PMC6920072

[80] Saha P, Sisodia SS. Role of the gut microbiome in mediating sex-specific differences in the pathophysiology of alzheimer’s disease. Neurotherapeutics. 2024;21:6. 10.1016/j.neurot.2024.e00426. 10.1016/j.neurot.2024.e00426 PMC11585881 39054179

[81] Peters BA, Santoro N, Kaplan RC, Qi QB. Spotlight on the gut microbiome in menopause: current insights. Int J Womens Health. 2022;14:1059–72. 10.2147/Ijwh.S340491. 35983178 10.2147/IJWH.S340491 PMC9379122

[82] Wang HQ, Shi F, Zheng LH, Zhou WH, Mi BW, Wu SY, et al. Gut microbiota has the potential to improve health of menopausal women by regulating Estrogen. Front Endocrinol. 2025;16. Artn 156233210.3389/Fendo.2025.1562332. 10.3389/fendo.2025.1562332 PMC12183514 40551890

[83] Mosconi L, Berti V, Quinn C, McHugh P, Petrongolo G, Varsavsky I, et al. Sex differences in Alzheimer risk: brain imaging of endocrine vs chronologic aging. Neurology. 2017;89(13):1382–90. 10.1212/WNL.0000000000004425. 28855400 10.1212/WNL.0000000000004425 PMC5652968

[84] Valentino RJ, Van Bockstaele E. Opposing regulation of the locus coeruleus by corticotropin-releasing factor and opioids. Potential for reciprocal interactions between stress and opioid sensitivity. Psychopharmacology (Berl). 2001;158(4):331–42. 10.1007/s002130000673. 11797054 10.1007/s002130000673

[85] Qiao YJ, Guo J, Xiao Q, Wang J, Zhang XF, Liang XX, et al. A study on the differences in the gut microbiota and metabolism between male and female mice in different stress periods. Exp Biol Med. 2025;250. Artn 1020410.3389/Ebm.2025.10204. 10.3389/ebm.2025.10204 PMC11851196 40008145

[86] Bostick JW, Connerly TJ, Thron T, Needham BD, de Castro Fonseca M, Kaddurah-Daouk R, et al. Genotype and microbiome shape immunity in a sex-specific manner in mouse models of Alzheimer’s disease. Brain Behav Immun. 2025;129:1014–27. 10.1016/j.bbi.2025.07.028. 40738263 10.1016/j.bbi.2025.07.028 PMC12490345

[87] Caestecker S, Lescrauwaet E, Boon P, Carrette E, Raedt R, Vonck K. The locus coeruleus-noradrenergic system in the healthy and diseased brain: a narrative review. Eur J Neurol. 2025;32(9):e70337. 10.1111/ene.70337. 40905374 10.1111/ene.70337 PMC12409474

[88] Pamphlett R. Uptake of environmental toxicants by the locus ceruleus: a potential trigger for neurodegenerative, demyelinating and psychiatric disorders. Med Hypotheses. 2014;82(1):97–104. 10.1016/j.mehy.2013.11.016. 24315447 10.1016/j.mehy.2013.11.016

[89] Cohen Z, Molinatti G, Hamel E. Astroglial and vascular interactions of noradrenaline terminals in the rat cerebral cortex. J Cereb Blood Flow Metab. 1997;17(8):894–904. 10.1097/00004647-199708000-00008. 9290587 10.1097/00004647-199708000-00008

[90] Braniste V, Al-Asmakh M, Kowal C, Anuar F, Abbaspour A, Tóth M, et al. The gut microbiota influences blood-brain barrier permeability in mice. Sci Transl Med. 2014;6:263. ARTN 263ra15810.1126/scitranslmed.3009759. 10.1126/scitranslmed.3009759 PMC4396848 25411471

[91] Zhang Y, Zhang S, Li BL, Luo YC, Gong YT, Jin XX, et al. Gut microbiota dysbiosis promotes age-related atrial fibrillation by lipopolysaccharide and glucose-induced activation of NLRP3-inflammasome. Cardiovasc Res. 2022;118(3):785–97. 10.1093/cvr/cvab114. 33757127 10.1093/cvr/cvab114

[92] Pan Z, Jia Z, Jiang T, Cai Q, Di Z, Gan L, et al. Modulation of the neurovascular unit by the locus coeruleus-norepinephrine system: from physiological mechanisms to therapeutic applications. FASEB J. 2025;39(20):e71127. 10.1096/fj.202502069R. 41078309 10.1096/fj.202502069R PMC12516802

[93] Finley KH, Cobb S. The capillary bed of the locus coeruleus. J Comp Neurol. 1940;73(1):49–58. 10.1002/cne.900730105.

[94] Edvinsson L, Nielsen KC, Owman C, West KA. Sympathetic neural influence on norepinephrine vasoconstriction in brain vessels. Arch Neurol. 1972;27(6):492–5. 10.1001/archneur.1972.00490180028007. 4563449 10.1001/archneur.1972.00490180028007

[95] Korte N, James G, You H, Hirunpattarasilp C, Christie I, Sethi H, et al. Noradrenaline released from locus coeruleus axons contracts cerebral capillary pericytes via alpha(2) adrenergic receptors. J Cereb Blood Flow Metab. 2023;43:7. 10.1177/0271678X231152549. 10.1177/0271678X231152549 PMC10291462 36688515

[96] Hayashi T, Yamada K, Esaki T, Kuzuya M, Satake S, Ishikawa T, et al. Estrogen increases endothelial nitric oxide by a receptor-mediated system. Biochem Biophys Res Commun. 1995;214(3):847–55. 10.1006/bbrc.1995.2364. 7575554 10.1006/bbrc.1995.2364

[97] Madison A, Kiecolt-Glaser JK. Stress, depression, diet, and the gut microbiota: human-bacteria interactions at the core of psychoneuroimmunology and nutrition. Curr Opin Behav Sci. 2019;28:105–10. 10.1016/j.cobeha.2019.01.011. 32395568 10.1016/j.cobeha.2019.01.011 PMC7213601

[98] Strehlow K, Rotter S, Wassmann S, Adam O, Grohe C, Laufs K, et al. Modulation of antioxidant enzyme expression and function by Estrogen. Circ Res. 2003;93:2:170–7. 10.1161/01.RES.0000082334.17947.11. 12816884 10.1161/01.RES.0000082334.17947.11

[99] Kumar S, Sharma S, Aggarwal S, Rafikov R, Noonepalle SK, Stepp DW, et al. Endothelial nitric oxide synthase uncoupling in a chemically induced model of menopause is associated with the development of endothelial dysfunction. Endocr Rev. 2010;31:3. ://WOS:000281989401329.

[100] Taddei S, Virdis A, Ghiadoni L, Mattei P, Sudano I, Bernini G, et al. Menopause is associated with endothelial dysfunction in women. Hypertension. 1996;28(4):576–82. 10.1161/01.Hyp.28.4.576. 8843881 10.1161/01.hyp.28.4.576

[101] Yung LM, Wong WT, Tian XY, Leung FP, Yung LH, Chen ZY et al. Inhibition of Renin-Angiotensin System Reverses Endothelial Dysfunction and Oxidative Stress in Estrogen Deficient Rats. Plos One. 2011;6:3; doi:ARTN e1743710.1371/journal.pone.0017437. 10.1371/journal.pone.0017437 PMC3066200 21479266

[102] Somani YB, Pawelczyk JA, De Souza MJ, Kris-Etherton PM, Proctor DN. Aging women and their endothelium: probing the relative role of estrogen on vasodilator function. Am J Physiol Heart Circ Physiol. 2019;317(2):H395-404. 10.1152/ajpheart.00430.2018. 31173499 10.1152/ajpheart.00430.2018 PMC6732482

[103] Keteepe-Arachi T, Sharma S. Cardiovascular disease in women: understanding symptoms and risk factors. Eur Cardiol. 2017;12:110–3. 10.15420/ecr.2016:32:1. 30416543 10.15420/ecr.2016:32:1 PMC6206467

[104] Sanchez-Padilla J, Guzman JN, Ilijic E, Kondapalli J, Galtieri DJ, Yang B, et al. Mitochondrial oxidant stress in locus coeruleus is regulated by activity and nitric oxide synthase. Nat Neurosci. 2014;17(6):832–40. 10.1038/nn.3717. 24816140 10.1038/nn.3717 PMC4131291

[105] Iannitelli AF, Weinshenker D. Riddles in the dark: decoding the relationship between neuromelanin and neurodegeneration in locus coeruleus neurons. Neurosci Biobehav Rev. 2023;152:105287. 10.1016/j.neubiorev.2023.105287. 37327835 10.1016/j.neubiorev.2023.105287 PMC10523397

[106] Zecca L, Zucca FA, Wilms H, Sulzer D. Neuromelanin of the substantia nigra: a neuronal black hole with protective and toxic characteristics. Trends Neurosci. 2003;26(11):578–80. 10.1016/j.tins.2003.08.009. 14585596 10.1016/j.tins.2003.08.009

[107] Moreno-Garcia A, Kun A, Calero M, Calero O. The neuromelanin paradox and its dual role in oxidative stress and neurodegeneration. Antioxidants. 2021. 10.3390/antiox10010124. 33467040 10.3390/antiox10010124 PMC7829956

[108] Kovats S. Estrogen receptors regulate innate immune cells and signaling pathways. Cell Immunol. 2015;294(2):63–9. 10.1016/j.cellimm.2015.01.018. 25682174 10.1016/j.cellimm.2015.01.018 PMC4380804

[109] Bouman A, Heineman MJ, Faas MM. Sex hormones and the immune response in humans. Hum Reprod Update. 2005;11(4):411–23. 10.1093/humupd/dmi008. 15817524 10.1093/humupd/dmi008

[110] Baker JM, Al-Nakkash L, Herbst-Kralovetz MM. Estrogen-gut microbiome axis: physiological and clinical implications. Maturitas. 2017;103:45–53. 10.1016/j.maturitas.2017.06.025. 28778332 10.1016/j.maturitas.2017.06.025

[111] Gall WE, Zawada G, Mojarrabi B, Tephly TR, Green MD, Coffman BL, et al. Differential glucuronidation of bile acids, androgens and estrogens by human UGT1A3 and 2B7. J Steroid Biochem Mol Biol. 1999;70(1–3):101–8. 10.1016/s0960-0760(99)00088-6. 10529008 10.1016/s0960-0760(99)00088-6

[112] Cheng Z, Rios GR, King CD, Coffman BL, Green MD, Mojarrabi B, et al. Glucuronidation of catechol estrogens by expressed human UDP-glucuronosyltransferases (UGTs) 1A1, 1A3, and 2B7. Toxicol Sci. 1998;45:152–7. 10.1006/toxs.1998.2494. 9848110 10.1006/toxs.1998.2494

[113] Plottel CS, Blaser MJ. Microbiome and malignancy. Cell Host Microbe. 2011;10(4):324–35. 10.1016/j.chom.2011.10.003. 22018233 10.1016/j.chom.2011.10.003 PMC3264051

[114] Flores R, Shi J, Fuhrman B, Xu X, Veenstra TD, Gail MH, et al. Fecal microbial determinants of fecal and systemic estrogens and estrogen metabolites: a cross-sectional study. J Transl Med. 2012;10:253. 10.1186/1479-5876-10-253. 23259758 10.1186/1479-5876-10-253 PMC3552825

[115] Hu SW, Ding QY, Zhang W, Kang MJ, Ma J, Zhao LH. Gut microbial beta-glucuronidase: a vital regulator in female estrogen metabolism. Gut Microbes. 2023;15:1 (doi:Artn 223674910.1080/19490976.2023.2236749). 10.1080/19490976.2023.2236749 PMC10416750 37559394

[116] Shughrue PJ, Lane MV, Merchenthaler I. Comparative distribution of estrogen receptor-alpha and -beta mRNA in the rat central nervous system. J Comp Neurol. 1997;388(4):507–25. 10.1002/(sici)1096-9861(19971201)388:4<507::aid-cne1>3.0.CO;2-6. 9388012

[117] Serova LI, Maharjan S, Huang A, Sun D, Kaley G, Sabban EL. Response of tyrosine hydroxylase and GTP cyclohydrolase I gene expression to estrogen in brain catecholaminergic regions varies with mode of administration. Brain Res. 2004;1015(1–2):1–8. 10.1016/j.brainres.2004.04.002. 15223360 10.1016/j.brainres.2004.04.002

[118] Parvez S, Ismahan G, Raza-Bukhari A, Youdim MB. Activity of catechol-o-methyltransferase in brain regions and adrenal gland during the oestrus cycle. J Neural Transm. 1978;42:4305–12. 10.1007/BF01673554. 10.1007/BF01673554 567245

[119] Tahri A, Amedei A. Unraveling the links between estrogen and gut microbiota in sex-hormone driven cancers. World J Clin Oncol. 2025;16:9 (doi:Artn 10881910.5306/Wjco.V16.I9.108819). 10.5306/wjco.v16.i9.108819 PMC12476589 41024833

[120] Peters BA, Lin J, Qi QB, Usyk M, Isasi CR, Mossavar-Rahmani Y, et al. Menopause is associated with an altered gut microbiome and estrobolome, with implications for adverse cardiometabolic risk in the Hispanic Community Health Study/Study of Latinos. mSystems. 2022. 10.1128/msystems.00273-22. 35675542 10.1128/msystems.00273-22 PMC9239235

[121] Zuloaga DG, Lafrican JJ, Zuloaga KL. Androgen regulation of behavioral stress responses and the hypothalamic-pituitary-adrenal axis. Horm Behav. 2024;162; doi:Artn 10552810.1016/J.Yhbeh.2024.105528. 10.1016/j.yhbeh.2024.105528 PMC11144109 38503191

[122] Oyola MG, Handa RJ. Hypothalamic-pituitary-adrenal and hypothalamic-pituitary-gonadal axes: sex differences in regulation of stress responsivity. Stress Int J Biol Stress. 2017;20:5476–94. 10.1080/10253890.2017.1369523. 10.1080/10253890.2017.1369523 PMC5815295 28859530

[123] Hamson DK, Jones BA, Watson NV. Distribution of androgen receptor immunoreactivity in the brainstem of male rats. Neuroscience. 2004;127(4):797–803. 10.1016/j.neuroscience.2004.06.006. 15312892 10.1016/j.neuroscience.2004.06.006

[124] Handa RJ, Nunley KM, Lorens SA, Louie JP, McGivern RF, Bollnow MR. Androgen regulation of adrenocorticotropin and corticosterone secretion in the male rat following novelty and foot shock stressors. Physiol Behav. 1994;55:1117–24. 10.1016/0031-9384(94)90018-3. 10.1016/0031-9384(94)90018-3 8140154

[125] Henderson VW, Brinton RD. Menopause and mitochondria: windows into estrogen effects on Alzheimer’s disease risk and therapy. Neuroendocrinology: Pathological Situations and Diseases. 2010;182:77–96. 10.1016/S0079-6123(10)82003-5. 10.1016/S0079-6123(10)82003-5 PMC5776041 20541661

[126] Janitzky K. Impaired phasic discharge of locus coeruleus neurons based on persistent high tonic discharge-a new hypothesis with potential implications for neurodegenerative diseases. Front Neurol. 2020;11:371. 10.3389/fneur.2020.00371. 32477246 10.3389/fneur.2020.00371 PMC7235306

[127] Jeong HG, Park H. Metabolic disorders in menopause. Metabolites. 2022. 10.3390/metabo12100954. 36295856 10.3390/metabo12100954 PMC9606939

[128] Sekhar TV, Medarametla S, Rahman A, Adapa SS. Early menopause in type 2 diabetes - a study from a South Indian tertiary care centre. J Clin Diagn Res. 2015;9(10):OC08-10. 10.7860/JCDR/2015/14181.6628. 26557555 10.7860/JCDR/2015/14181.6628 PMC4625274

[129] Monterrosa-Castro A, Portela-Buelvas K, Blumel-Mendez JE. Early and premature menopause in women with diabetes mellitus type 2. Expert Rev Endocrinol Metab. 2014;9(4):297–9. 10.1586/17446651.2014.922863. 30763989 10.1586/17446651.2014.922863

[130] Groves DA, Bowman EM, Brown VJ. Recordings from the rat locus coeruleus during acute vagal nerve stimulation in the anaesthetised rat. Neurosci Lett. 2005;379(3):174–9. 10.1016/j.neulet.2004.12.055. 15843058 10.1016/j.neulet.2004.12.055

[131] Hulsey DR, Riley JR, Loerwald KW, Rennaker RL 2nd, Kilgard MP, Hays SA. Parametric characterization of neural activity in the locus coeruleus in response to vagus nerve stimulation. Exp Neurol. 2017;289:21–30. 10.1016/j.expneurol.2016.12.005. 27988257 10.1016/j.expneurol.2016.12.005 PMC5297969

[132] Goehler LE, Gaykema RP, Hansen MK, Anderson K, Maier SF, Watkins LR. Vagal immune-to-brain communication: a visceral chemosensory pathway. Auton Neurosci. 2000;85(1–3):49–59. 10.1016/S1566-0702(00)00219-8. 11189026 10.1016/S1566-0702(00)00219-8

[133] Watkins LR, Maier SF, Goehler LE. Cytokine-to-brain communication: a review & analysis of alternative mechanisms. Life Sci. 1995;57(11):1011–26. 10.1016/0024-3205(95)02047-m. 7658909 10.1016/0024-3205(95)02047-m

[134] Valentino RJ, Van Bockstaele E. Convergent regulation of locus coeruleus activity as an adaptive response to stress. Eur J Pharmacol. 2008;583(2–3):194–203. 10.1016/j.ejphar.2007.11.062. 18255055 10.1016/j.ejphar.2007.11.062 PMC2349983

[135] Reyes BAS. The locus coeruleus: Anatomy, Physiology, and Stress-Related neuropsychiatric disorders. Eur J Neurosci. 2025;61(7). ARTN e7011110.1111/ejn.70111. 10.1111/ejn.70111 PMC11992612 40219735

[136] Goehler LE, Gaykema RP, Nguyen KT, Lee JE, Tilders FJ, Maier SF, et al. Interleukin-1beta in immune cells of the abdominal vagus nerve: a link between the immune and nervous systems? J Neurosci. 1999;19:72799–806. 10.1523/JNEUROSCI.19-07-02799.1999. 10.1523/JNEUROSCI.19-07-02799.1999 PMC6786076 10087091

[137] Marquette C, Linard C, Galonnier M, Van Uye A, Mathieu J, Gourmelon P, et al. IL-1beta, TNFalpha and IL-6 induction in the rat brain after partial-body irradiation: role of vagal afferents. Int J Radiat Biol. 2003;79(10):777–85. 10.1080/09553000310001610998. 14630536 10.1080/09553000310001610998

[138] Aston-Jones G, Cohen JD. An integrative theory of locus coeruleus-norepinephrine function: adaptive gain and optimal performance. Annu Rev Neurosci. 2005;28:403–50. 10.1146/annurev.neuro.28.061604.135709. 16022602 10.1146/annurev.neuro.28.061604.135709

[139] Harley CW, Yuan Q. Locus coeruleus optogenetic modulation: lessons learned from temporal patterns. Brain Sci. 2021. 10.3390/brainsci11121624. 34942924 10.3390/brainsci11121624 PMC8699422

[140] Rios-Covian D, Gonzalez S, Nogacka AM, Arboleya S, Salazar N, Gueimonde M, et al. An overview on fecal branched short-chain fatty acids along human life and as related with body mass index: associated dietary and anthropometric factors. Front Microbiol. 2020;11:973. 10.3389/fmicb.2020.00973. 32547507 10.3389/fmicb.2020.00973 PMC7271748

[141] Lal S, Kirkup AJ, Brunsden AM, Thompson DG, Grundy D. Vagal afferent responses to fatty acids of different chain length in the rat. Am J Physiol Gastrointest Liver Physiol. 2001;281(4):G907–15. 10.1152/ajpgi.2001.281.4.G907. 11557510 10.1152/ajpgi.2001.281.4.G907

[142] Diz-Chaves Y, Herrera-Perez S, Gonzalez-Matias LC, Lamas JA, Mallo F. Glucagon-Like Peptide-1 (GLP-1) in the integration of neural and endocrine responses to stress. Nutrients. 2020. 10.3390/nu12113304. 33126672 10.3390/nu12113304 PMC7692797

[143] Barakat GM, Ramadan W, Assi G, Khoury NBE. Satiety: a gut-brain-relationship. J Physiol Sci. 2024;74(1):11. 10.1186/s12576-024-00904-9. 38368346 10.1186/s12576-024-00904-9 PMC10874559

[144] Kratsman N, Getselter D, Elliott E. Sodium butyrate attenuates social behavior deficits and modifies the transcription of inhibitory/excitatory genes in the frontal cortex of an autism model. Neuropharmacology. 2016;102:136–45. 10.1016/j.neuropharm.2015.11.003. 26577018 10.1016/j.neuropharm.2015.11.003

[145] Dalile B, Van Oudenhove L, Vervliet B, Verbeke K. The role of short-chain fatty acids in microbiota-gut-brain communication. Nat Rev Gastroenterol Hepatol. 2019;16(8):461–78. 10.1038/s41575-019-0157-3. 31123355 10.1038/s41575-019-0157-3

[146] Liu CC, Kuo TB, Yang CC. Effects of estrogen on gender-related autonomic differences in humans. Am J Physiol Heart Circ Physiol. 2003;285(5):H2188-93. 10.1152/ajpheart.00256.2003. 12881217 10.1152/ajpheart.00256.2003

[147] Qiao GF, Li BY, Lu YJ, Fu YL, Schild JH. 17Beta-estradiol restores excitability of a sexually dimorphic subset of myelinated vagal afferents in ovariectomized rats. Am J Physiol Cell Physiol. 2009;297(3):C654-64. 10.1152/ajpcell.00059.2009. 19570896 10.1152/ajpcell.00059.2009 PMC2740394

[148] Li BY, Qiao GF, Feng B, Zhao RB, Lu YJ, Schild JH. Electrophysiological and neuroanatomical evidence of sexual dimorphism in aortic baroreceptor and vagal afferents in rat. Am J Physiol Regul Integr Comp Physiol. 2008;295:R41301–10. 10.1152/ajpregu.90401.2008. 10.1152/ajpregu.90401.2008 PMC2576098 18685060

[149] Biscola NP, Bartmeyer PM, Beshay Y, Stern E, Mihaylov PV, Powley TL, et al. Laterality, sexual dimorphism, and human vagal projectome heterogeneity shape neuromodulation to vagus nerve stimulation. Commun Biol. 2024;7(1):1536. 10.1038/s42003-024-07222-1. 39562711 10.1038/s42003-024-07222-1 PMC11576867

[150] Coolen RL, Cambier JC, Spantidea PI, van Asselt E, Blok BFM. Androgen receptors in areas of the spinal cord and brainstem: a study in adult male cats. J Anat. 2021;239(1):125–35. 10.1111/joa.13407. 33619726 10.1111/joa.13407 PMC8197961

[151] Sun P, Wang J, Zhang M, Duan X, Wei Y, Xu F, et al. Sex-related differential whole-brain input atlas of locus coeruleus noradrenaline neurons. Front Neural Circuits. 2020;14:53. 10.3389/fncir.2020.00053. 33071759 10.3389/fncir.2020.00053 PMC7541090

[152] Bedford A, Gong J. Implications of butyrate and its derivatives for gut health and animal production. Anim Nutr. 2018;4(2):151–9. 10.1016/j.aninu.2017.08.010. 30140754 10.1016/j.aninu.2017.08.010 PMC6104520

[153] Namkung H, Yu H, Gong J, Leeson S. Antimicrobial activity of butyrate glycerides toward *Salmonella* Typhimurium and *Clostridium perfringens*. Poult Sci. 2011;90(10):2217–22. 10.3382/ps.2011-01498. 21934003 10.3382/ps.2011-01498

[154] Den Hond E, Hiele M, Evenepoel P, Peeters M, Ghoos Y, Rutgeerts P. In vivo butyrate metabolism and colonic permeability in extensive ulcerative colitis. Gastroenterology. 1998;115(3):584–90. 10.1016/s0016-5085(98)70137-4. 9721155 10.1016/s0016-5085(98)70137-4

[155] Hou Y, Li J, Ying S. Tryptophan metabolism and gut microbiota: a novel regulatory axis integrating the microbiome, immunity, and cancer. Metabolites. 2023;13(11):1166. 10.3390/metabo13111166. 37999261 10.3390/metabo13111166 PMC10673612

[156] Fogelson KA, Dorrestein PC, Zarrinpar A, Knight R. The gut microbial bile acid modulation and its relevance to digestive health and diseases. Gastroenterology. 2023;164(7):1069–85. 10.1053/j.gastro.2023.02.022. 36841488 10.1053/j.gastro.2023.02.022 PMC10205675

[157] Jenkins TA, Nguyen JC, Polglaze KE, Bertrand PP. Influence of tryptophan and serotonin on mood and cognition with a possible role of the gut-brain axis. Nutrients. 2016. 10.3390/nu8010056. 26805875 10.3390/nu8010056 PMC4728667

[158] Xing C, Huang X, Wang D, Yu D, Hou S, Cui H, et al. Roles of bile acids signaling in neuromodulation under physiological and pathological conditions. Cell Biosci. 2023;13(1):106. 10.1186/s13578-023-01053-z. 37308953 10.1186/s13578-023-01053-z PMC10258966

[159] Mulak A. Bile acids as key modulators of the Brain-Gut-Microbiota axis in Alzheimer’s disease. J Alzheimers Dis. 2021;84:2461–77. 10.3233/Jad-210608. 10.3233/JAD-210608 PMC8673511 34569953

[160] O’Mahony SM, Clarke G, Borre YE, Dinan TG, Cryan JF. Serotonin, tryptophan metabolism and the brain-gut-microbiome axis. Behav Brain Res. 2015;277:32–48. 10.1016/j.bbr.2014.07.027. 25078296 10.1016/j.bbr.2014.07.027

[161] Duboc H, Tache Y, Hofmann AF. The bile acid TGR5 membrane receptor: from basic research to clinical application. Dig Liver Dis. 2014;46(4):302–12. 10.1016/j.dld.2013.10.021. 24411485 10.1016/j.dld.2013.10.021 PMC5953190

[162] Pols TW, Noriega LG, Nomura M, Auwerx J, Schoonjans K. The bile acid membrane receptor TGR5 as an emerging target in metabolism and inflammation. J Hepatol. 2011;54(6):1263–72. 10.1016/j.jhep.2010.12.004. 21145931 10.1016/j.jhep.2010.12.004 PMC3650458

[163] Feng L, Zhou N, Li Z, Fu D, Guo Y, Gao X, et al. Co-occurrence of gut microbiota dysbiosis and bile acid metabolism alteration is associated with psychological disorders in Crohn’s disease. FASEB J. 2022;36(1):e22100. 10.1096/fj.202101088RRR. 34939244 10.1096/fj.202101088RRR

[164] Tao YL, Zhou HY, Li ZK, Wu H, Wu FG, Miao ZG, et al. TGR5 deficiency-induced anxiety and depression-like behaviors: the role of gut microbiota dysbiosis. J Affect Disord. 2024;344:219–32. 10.1016/j.jad.2023.10.072. 37839469 10.1016/j.jad.2023.10.072

[165] Klindt C, Reich M, Hellwig B, Stindt J, Rahnenführer J, Hengstler JG, et al. The G Protein-Coupled bile acid receptor TGR5 (Gpbar1) modulates Endothelin-1 signaling in liver. Cells. 2019;8:11. doi:Artn 146710.3390/Cells8111467. 10.3390/cells8111467 PMC6912679 31752395

[166] Sinha SR, Haileselassie Y, Nguyen LP, Tropini C, Wang M, Becker LS, et al. Dysbiosis-Induced secondary bile acid deficiency promotes intestinal inflammation. Cell Host Microbe. 2020;27. 10.1016/j.chom.2020.01.021. 4:659 – 70 e5. 10.1016/j.chom.2020.01.021 PMC8172352 32101703

[167] Zangerolamo L, Carvalho M, Barbosa HCL. The critical role of the bile acid receptor TGR5 in energy homeostasis: insights into physiology and therapeutic potential. Int J Mol Sci. 2025;26(14):6547. 10.3390/Ijms26146547. 40724796 10.3390/ijms26146547 PMC12294878

[168] Gao K, Mu CL, Farzi A, Zhu WY. Tryptophan metabolism: a link between the gut microbiota and brain. Adv Nutr. 2020;11:3709–23. 10.1093/advances/nmz127. 10.1093/advances/nmz127 PMC7231603 31825083

[169] Lin P, Li D, Shi Y, Li QT, Guo XK, Dong K, et al. Dysbiosis of the gut microbiota and kynurenine (Kyn) pathway activity as potential biomarkers in patients with major depressive disorder. Nutrients. 2023;15(7):1752. 10.3390/Nu15071752. 37049591 10.3390/nu15071752 PMC10096701

[170] Szabo ST, Blier P. Functional and pharmacological characterization of the modulatory role of serotonin on the firing activity of locus coeruleus norepinephrine neurons. Brain Res. 2001;922(1):9–20. 10.1016/S0006-8993(01)03121-3. 11730697 10.1016/s0006-8993(01)03121-3

[171] Bennion LJ, Drobny E, Knowler WC, Ginsberg RL, Garnick MB, Adler RD, et al. Sex differences in the size of bile acid pools. Metabolism. 1978;27(8):961–9. 10.1016/0026-0495(78)90140-3. 672615 10.1016/0026-0495(78)90140-3

[172] Turley SD, Schwarz M, Spady DK, Dietschy JM. Gender-related differences in bile acid and sterol metabolism in outbred CD-1 mice fed low- and high-cholesterol diets. Hepatology. 1998;28(4):1088–94. 10.1002/hep.510280425. 9755247 10.1002/hep.510280425

[173] Li-Hawkins J, Gåfvels M, Olin M, Lund EG, Andersson U, Schuster G, et al. Cholic acid mediates negative feedback regulation of bile acid synthesis in mice. J Clin Invest. 2002;110:81191–200. 10.1172/Jci200216309. 10.1172/JCI16309 PMC150802 12393855

[174] Zu Y, Yang J, Zhang C, Liu D. The pathological mechanisms of estrogen-induced cholestasis: current perspectives. Front Pharmacol. 2021;12:761255. 10.3389/fphar.2021.761255. 34819862 10.3389/fphar.2021.761255 PMC8606790

[175] Ovadia C, Perdones-Montero A, Spagou K, Smith A, Sarafian MH, Gomez-Romero M, et al. Enhanced microbial bile acid deconjugation and impaired ileal uptake in pregnancy repress intestinal regulation of bile acid synthesis. Hepatology. 2019;70(1):276–93. 10.1002/hep.30661. 30983011 10.1002/hep.30661 PMC6619257

[176] Phelps T, Snyder E, Rodriguez E, Child H, Harvey P. The influence of biological sex and sex hormones on bile acid synthesis and cholesterol homeostasis. Biology of sex differences. 2019;10:1; doi:ARTN 5210.1186/s13293-019-0265-3. 10.1186/s13293-019-0265-3 PMC6880483 31775872

[177] Lejri I, Grimm A, Eckert A. Mitochondria, estrogen and female brain aging. Front Aging Neurosci. 2018;10:124. 10.3389/fnagi.2018.00124. 29755342 10.3389/fnagi.2018.00124 PMC5934418

[178] Ruo SW, Alkayyali T, Win M, Tara A, Joseph C, Kannan A, et al. Role of gut microbiota dysbiosis in breast cancer and novel approaches in Prevention, Diagnosis, and treatment. Cureus. 2021;13:8e17472. 10.7759/cureus.17472. 10.7759/cureus.17472 PMC8405251 34513524

[179] Nebel RA, Aggarwal NT, Barnes LL, Gallagher A, Goldstein JM, Kantarci K, et al. Understanding the impact of sex and gender in Alzheimer’s disease: a call to action. Alzheimers Dement. 2018;14(9):1171–83. 10.1016/j.jalz.2018.04.008. 29907423 10.1016/j.jalz.2018.04.008 PMC6400070

